# Comprehensive reference ranges for cardiovascular magnetic resonance: time to move on from single centre data?

**DOI:** 10.1007/s10554-025-03370-5

**Published:** 2025-03-27

**Authors:** John P. Farrant, Nicholas Black, Kentaro Yamagata, Fardad Soltani, Christopher Orsborne, Chi Kit Yan, David Clark, Luke Pleva, Clifford Garratt, Matthias Schmitt, Bernard Clarke, Josephine Naish, Anna Reid, Christopher A. Miller

**Affiliations:** 1 Division of Cardiovascular Sciences, Faculty of Biology, School of Medical Sciences, Medicine and Health, Manchester Academic Health Science Centre, University of Manchester, Oxford Road, Manchester, M13 9PL United Kingdom; 2 Manchester University NHS Foundation Trust, Wythenshawe Hospital, Manchester, M23 9LT United Kingdom; 3 Division of Cell-Matrix Biology & Regenerative Medicine, Faculty of Biology, Wellcome Centre for Cell-Matrix Research, School of Biology, Medicine and Health, Manchester Academic Health Science Centre, University of Manchester, Oxford Road, Manchester, M13 9PT United Kingdom

**Keywords:** Normative values, Comprehensive reference ranges, Interscan reproducibility, Common variations in practice, Cardiovascular magnetic resonance

## Abstract

Cardiovascular magnetic resonance (CMR) provides gold standard, and often unique, measurements of cardiovascular structure, function and tissue character. Fundamental to such capabilities are clearly defined normal ranges. This study aimed to (1) Determine normal ranges for an extensive set of CMR measurements, and the inter-scan reproducibility of these measurements; (2) Determine the impact of common variations in practice, and; (3) Systematically evaluate the findings in the context of published reference ranges. One hundred and 22 healthy adults, including a minimum of 10 males and 10 females per age decile, underwent assessment including CMR (3 T, Siemens). Twenty participants returned for a second CMR. Image analysis was performed using cvi42 by experienced observers. Age- and sex-specific reference ranges, in tabular and normogram formats, and their interscan reproducibility, are provided for left ventricular mass, wall thickness, volumes and ejection fraction; right ventricular volumes and ejection fraction; longitudinal, radial and circumferential LV strains; atrial area, volume and strains; native T1, T2, T2*, aortic distensibility and pulse wave velocity. Measurement reproducibility improved when baseline scans were used for reference, e.g., for basal slice selection. Myocardial T1 was the most reproducible of all CMR measurements. Common variations in practice resulted in significant measurement differences e.g., indexed left atrial volume was larger (47.3 vs 40.3 ml/m^2^, *P* < 0.0001), and its measurement less variable, when measured from atrial short-axis cine stacks compared to biplanar measurement from 4- to 2-chamber cines. Studies using similar methods to define normal ranges demonstrate clinically-relevant differences in the normal ranges produced. A comprehensive set of age and sex specific CMR reference ranges are provided, along with inter-scan reproducibility and the impact of common variations in practice. Single centre studies, whilst meticulous in design and delivery, result in clinically-relevant variations in normal ranges. We advocate that larger cohorts, including diverse ethnicities, such as the Healthy Hearts Consortium, may be a better approach to defining normal ranges for common CMR measurements.

**Graphical abstract:**

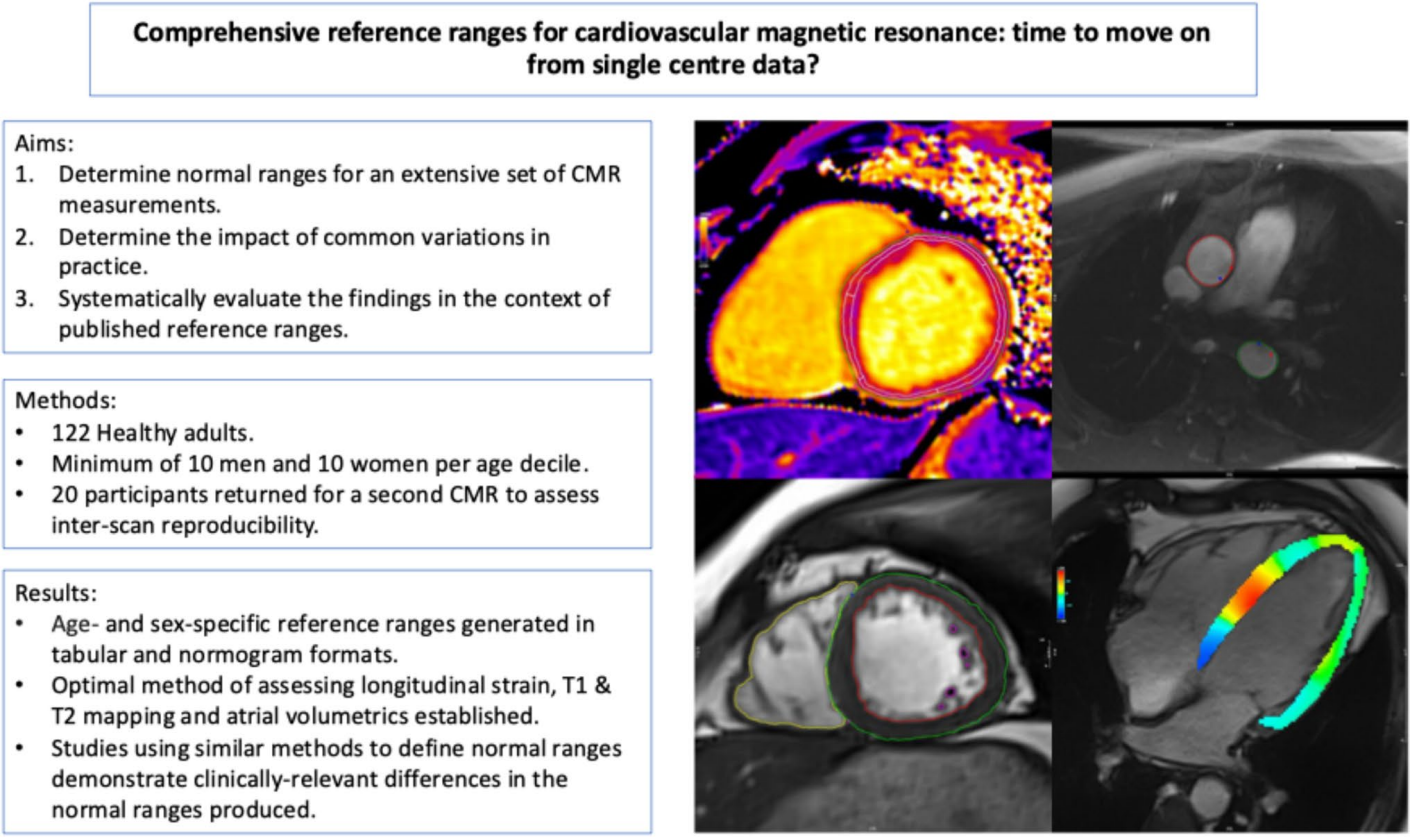

**Supplementary Information:**

The online version contains supplementary material available at 10.1007/s10554-025-03370-5.

## Introduction

Cardiovascular magnetic resonance (CMR) provides gold standard, and often unique, measurements of cardiovascular structure, function and tissue character that are central to the contemporary diagnosis and management of patients with cardiovascular disease. Fundamental to such capabilities are clearly defined normal ranges.

Before the widespread uptake of clinical CMR, the preeminent works by Maceira et al. and Hudsmith et al. provided the field with rigorously defined reference ranges for measurements of cardiac chamber mass, size and function [1–5]. The Maceira et al. data remain the basis for the European Association of Cardiovascular Imaging CMR reference ranges, which are widely used in clinical practice [6]. Such papers also provided a template for studies seeking to define adult normal ranges, for these and for more complex measurements, typically including around 10 males and females per age decile from a single centre.

In their 2020 updated review, Kawel‐Boehm et al. highlighted the small numbers of patients from which reference ranges had been derived at that stage [7]. For example, for the anatomically-correct method where papillary muscles are included in left ventricular (LV) mass, data from only 34 males aged 60 or over had contributed to normal ranges for LV volumetrics, ejection fraction (EF) and mass; data from only 10 females from each age decile had contributed to normal ranges for equivalent right ventricular (RV) measurements. This paper, and others, also serve to demonstrate the variability in reported normal ranges, outwith the well described impact of anatomically correct versus smooth contouring. Recently, using data from six countries, albeit predominantly (85%) from UK Biobank, the Healthy Hearts Consortium have defined normal reference ranges for ventricular volumetrics, EF and mass, and atrial volumetrics, using a much larger cohort [8]. Reference ranges for other measurements, such as aortic function, ventricular and atrial strain and parametric mapping, and the impact of common variations in their measurement, are less well defined [9, 10].

In this study we aimed to (1) Determine normal ranges for an extensive set of CMR measurements and the inter-scan reproducibility of these measurements; (2) Determine the impact of common variations in practice, and; (3). Systematically evaluate the findings in the context of published reference ranges.

## Methods

### Study design

Normal MCMR (Normative cardiovascular magnetic resonance values for measurement of cardiovascular structure and function at the Manchester Centre for Heart and Lung Magnetic Resonance Research) was an observational study designed to determine normal ranges for CMR measurements of cardiovascular structure, function and tissue character for people living in Greater Manchester, UK, and evaluate the findings in the context of published data. The study protocol is available as a supplemental appendix (Supplement 2). The study was sponsored by Manchester University NHS Foundation Trust, approved by a UK ethics committee (21/WA/0272), and registered on clinicaltrials.gov (NCT05066269). All participants provided written informed consent. The authors vouch for the accuracy and completeness of the data, and the fidelity of the study to the protocol.

### Participants

Volunteers were recruited via poster advertisement; adverts were placed in entrances, corridors, waiting areas and staff arears of Manchester NHS Foundation Trust Wythenshawe campus. Eligibility criteria included age over 18, no known history of cardiovascular disease and a normal resting 12-lead ECG. Exclusion criteria included contraindications to MRI scanning. We aimed to recruit 10 males and 10 females per age decile, from ages 18 to 29 up to 70 and over.

### Study visits

The study visit comprised informed consent, medical history, measurement of height and weight, 12-lead electrocardiogram (ECG) and CMR. 20 participants returned for a second visit to allow evaluation of the reproducibility of CMR measurements. Height and weight were used to calculate the body surface area (BSA) using the Mosteller formula.

### CMR image acquisition and analysis

CMR was performed using a 3 T scanner (Magnetom Vida, Syngo MR XA20 version; Siemens Medical Solutions, Erlangen, Germany) with a body 18-channel receiver coil at the BHF Manchester Centre for Heart and Lung Magnetic Resonance Research, Manchester University NHS Foundation Trust, UK. The CMR protocol is provided in the supplemental appendix (Supplement 1). No contrast agent was administered. Total scan time was approximately 45 min. Image analysis was performed using cvi42 (version 5.12.1; Circle Cardiovascular Imaging; Calgary, AB, Canada) by a single experienced observer (JPF). Details are provided in the supplemental appendix.

**Fig. 1:**
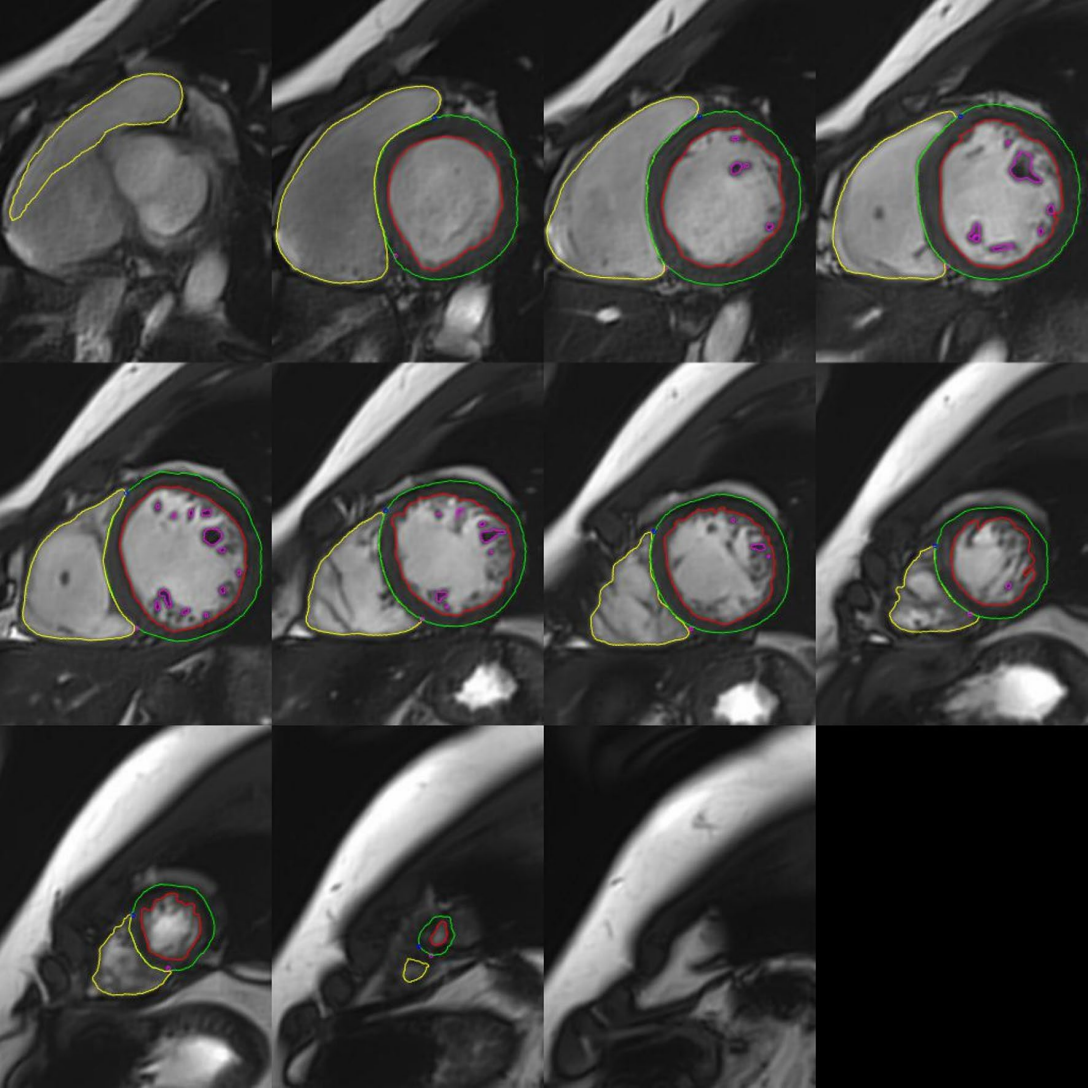
Example ventricular contouring. Papillary muscles and trabecular tissue were included in mass measurements and excluded from volume measurements (‘anatomically correct’)

### CMR measurements

1. LV mass, maximum wall thickness, volumes and ejection fraction. The ‘anatomically correct’ method of LV contouring was used for analysis, i.e., papillary muscles and trabecular tissue were included in measurement of LV mass (Fig. 1).
2. Two dimensional (2D) and three dimensional (3D) longitudinal, radial and circumferential LV strain. The impact of measuring LV global longitudinal strain from a 4-chamber cine versus measuring it from 4-, 2- and 3-chamber cines combined was evaluated.
3. Right ventricular (RV) volumes, ejection fraction and tricuspid annular plane systolic excursion (TAPSE). Papillary muscles and trabecular tissue were excluded from RV volumes.
4. Atrial diameter, area, volume and strain (peak, conduit and booster). The impact of measuring left atrial (LA) volume from 4- and 2-chamber cines using Simpson’s biplane method versus measuring it from an atrial short-axis cine stack using Simpson’s summation of discs method was evaluated.
5. Native myocardial T1 and T2 relaxation times. The impact of measuring myocardial T1 and T2 from a mid-ventricular slice versus basal- and mid-ventricular slices, and from septal versus global myocardium, were evaluated.
6. T2*
7. Pulmonary artery size
8. Ascending and descending aortic distensibility, time to 50% peak ascending and descending aortic flow, and pulse wave velocity.

(It is important to note that anatomically correct versus smooth endocardial contouring leads to well recognised differences in ventricular volumes, mass and ejection fraction. Whilst this is clearly important, it is in addition to the factors being investigated and discussed here; in the current study, only anatomically correct contouring was used, and comparisons are made only with other studies using anatomically correct contouring).

### Inter-scan reproducibility of CMR measurements

Twenty participants returned for a second visit, conducted a minimum of 1 week after the baseline visit. Baseline and follow-up scans were both analysed by the same observer (JPF), a minimum of 2 weeks apart to minimise the risk of bias. The reproducibility of ventricular volumes, EF and mass was evaluated using two methods to mimic clinical practice:

1. Baseline and follow-up scans were analysed independently.
2. Follow-up scans were analysed after checking basal ventricular identification was consistent with the baseline scan.

### Inter-observer variability

Twenty randomly selected subjects were analysed independently by two observers (JPF and KY).

### Statistical analysis

Statistical analysis was performed in R (version 4.2.1; R Foundation for Statistical Computing, Vienna, Austria). Numerical data are expressed as either mean ± standard deviation or mean and 95% confidence intervals. Coefficient of variation (CoV) and Bland Altman limits of agreement were calculated to assess reproducibility and inter-observer variability. Between group differences were assessed using independent *t*-tests or non-parametric equivalents. Normograms, including mean and 95% prediction intervals, were generated to illustrate the change in variables with age using simple linear regression. Restricted cubic splines with three knots were used to fit non-linear normograms. *P* values < 0.05 were considered significant.

## Results

### Baseline characteristics

Consent was provided by 124 volunteers, but 2 were unable to complete the CMR due to claustrophobia and were therefore excluded. 122 volunteers were included in the analysis, with 10 females and 10 males per age decile (except the female 50–59 decile, which included 12 females). Baseline characteristics are provided in Table 1.

**Table 1:** Baseline characteristics according to age decile

Males	18–29	30–39	40–49	50–59	60–69	70 +
BSA	1.99 ± 0.23	2.01 ± 0.16	1.93 ± 0.2	2.08 ± 0.26	2 ± 0.15	2 ± 0.21
BMI	24.9 ± 3.6	25 ± 3.3	25.5 ± 3.5	27.5 ± 5	27.1 ± 2.1	27.3 ± 4.2
Heart rate	71 ± 12	61 ± 8	63 ± 6	63 ± 14	68 ± 14	70 ± 13
Systolic BP	121 ± 19	117 ± 7	112 ± 8	120 ± 10	139 ± 17	140 ± 22
Diastolic BP	65 ± 5	66 ± 8	68 ± 5	73 ± 11	81 ± 12	72 ± 9
QRS Duration	101 ± 8	94 ± 6	96 ± 13	101 ± 7	94 ± 11	101 ± 10

Females	18–29	30–39	40–49	50–59	60–69	70 +
BSA	1.67 ± 0.2	1.8 ± 0.27	1.78 ± 0.18	1.69 ± 0.12	1.64 ± 0.16	1.72 ± 0.17
BMI	22.1 ± 3.4	26 ± 5.8	25.4 ± 5.3	24.6 ± 3.4	23.1 ± 2.6	27.9 ± 4
Heart rate	80 ± 20	69 ± 10	65 ± 7	69.7 ± 8.1	73 ± 8	68 ± 10
Systolic BP	104 ± 7	104 ± 7	117 ± 11	117 ± 17	128 ± 19	127 ± 16
Diastolic BP	60 ± 7	62 ± 5	69 ± 8	68 ± 11	66 ± 10	64 ± 10
QRS Duration	89 ± 5	91 ± 8	91 ± 9	97 ± 11	92 ± 7	89 ± 5

Mean ± standard deviation. *BMI* body mass index, *BP* blood pressure, *BSA* body surface area

### Ventricular mass, volumes and EF

**Fig. 2:**
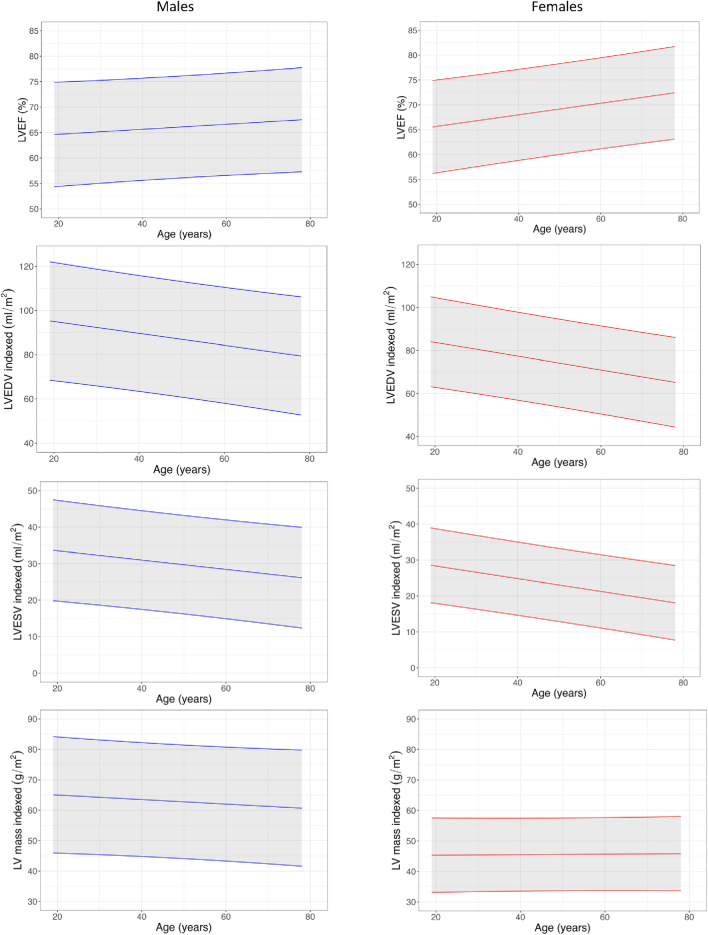
Left ventricular normograms

**Fig. 3:**
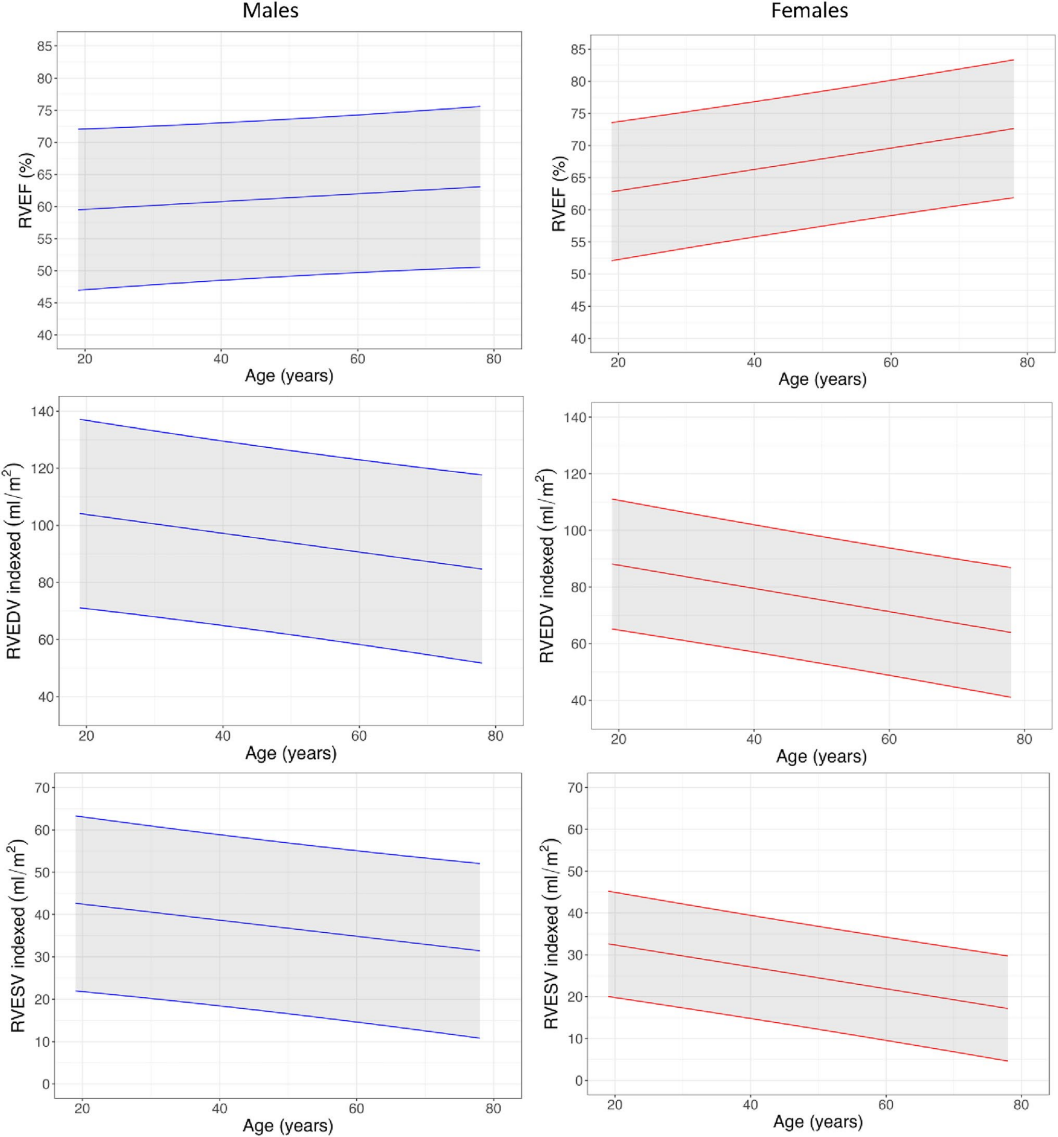
Right ventricular normograms

BSA-indexed LV and RV volumes, ejection fraction and mass measurements by sex and age are presented in Tables 2 and 3, Figs. 2 and 3, and Supplemental Tables 2 and 3. Ventricular volumes declined with age. Ejection fraction increased with age. Females had higher ejection fractions than males in all age deciles except for age under 30 years. Indexed LV mass (LVMi) was markedly higher in males than in females but did not change with age for either sex.

**Table 2:** Key male normal ranges according to age decile

	18–29	30–39	40–49	50–59	60–69	70 +
LVEF (%)	65 (57–72)	64 (53–76)	66 (59–73)	65 (52–77)	65 (52–78)	68 (58–78)
LVEDVi (ml/m^2^)	89 (77–101)	93 (77–108)	85 (65–104)	85 (60–111)	82 (53–111)	76 (45–106)
LVESVi (ml/m^2^)	32 (23–41)	33 (20–46)	29 (21–36)	30 (15–46)	29 (13–46)	24 (11–38)
LVMi (g/m^2^)	66 (44–87)	64 (50–79)	61 (41–81)	64 (49–79)	64 (43–86)	58 (43–72)
RVEF (%)	58 (46–69)	61 (49–73)	59 (53–65)	61 (47–75)	59 (44–75)	64 (50–78)
RVEDVi (ml/m^2^)	100 (74–125)	99 (74–124)	94 (65–123)	93 (59–127)	90 (58–122)	80 (49–111)
RVESVi (ml/m^2^)	43 (22–63)	40 (20–59)	39 (24–54)	38 (14–62)	37 (15–59)	29 (12–46)
LV global longitudinal strain (%)	− 17 (-22–13)	− 17 (-20–15)	− 17 (-19–15)	− 17 (-22–12)	− 16 (-20–13)	− 17 (-21–13)
LV global radial strain (%)	31 (19–42)	30 (23–37)	29 (24–33)	29 (16–43)	28 (19–37)	30 (18–42)
LV global circumferential strain (%)	− 18 (-22–14)	− 18 (-22–15)	− 18 (-20–15)	− 18 (-23–12)	− 18 (-20–15)	− 20 (-24–17)
LAVi (ml/m^2^)*	41 (18–64)	47 (28–65)	44 (30–57)	41 (22–59)	37 (5–68)	42 (13–71)
LA strain peak (%)	19.3 (14.3–24.2)	20.5 (15.3–25.6)	19.7 (15.2–24.2)	17.2 (11.9–22.5)	18.3 (14.2–22.5)	15.7 (9.8–21.6)
LA strain conduit (%)	13.5 (8.4–18.7)	12.6 (9.8–15.3)	12.2 (8–16.3)	10.3 (5.2–15.4)	9.4 (5.9–12.9)	8.3 (3.6–13)
LA strain booster (%)	7.3 (4.9–9.7)	9 (5.4–12.7)	8.7 (4.2–13.1)	8 (5.5–10.6)	9.3 (7.1–11.5)	8.2 (5.6–10.7)
LV native T1 (ms)**	1177 (1138–1217)	1190 (1168–1213)	1177 (1135–1220)	1194 (1144–1243)	1190 (1119–1261)	1197 (1103–1291)
LV native T2 (ms)**	38.4 (35.3–41.4)	38.2 (35.2–41.1)	38.8 (36.9–40.7)	39.4 (36.8–42.1)	38.9 (35.4–42.3)	39.8 (36.8–42.8)
LV septal T2-star (ms)	31 (24–38)	31.6 (24.3–38.9)	30.2 (23.7–36.7)	31.6 (16.8–46.5)	28 (20.5–35.5)	28.2 (15.6–40.8)
AAo distensibility (10^−3^ mmHg^−1^)	6.3 (3.6–9.1)	5.6 (2.5–8.7)	4.4 (1.2–7.5)	4.5 (1.4–7.7)	1.7 (0.6–2.9)	1.6 (0–3.3)
Pulse wave velocity (m/s)	4.1 (2.6–5.7)	4.4 (3–5.8)	5.2 (2.7–7.6)	6.3 (0.8–11.8)	9.6 (1.8–17.4)	11.3 (2.9–19.6)

Mean (95% Confidence Interval)

*LA volume obtained using biplanar method

** Obtained from basal and mid slices

*AAo* Ascending Aorta, *BSA* body surface area, *g/m*^2^, grams per metre squared, *LAVi* left atrial volume index, *LVEF* left ventricular ejection fraction, *LVEDVi* left ventricular end-diastolic volume index, *LVESVi* left ventricular end-systolic volume index, *LVMi* left ventricular mass index, *ml* millilitres, *ml/m*^2^ millilitres per metre squared, *mmHg* millimetres of mercury, *m/s* metres per second, *ms* milliseconds, *RVEF* right ventricular ejection fraction, *RVEDVi* right ventricular end-diastolic volume index, *RVESVi* right ventricular end-systolic volume index

**Table 3:** Key female normal ranges according to age decile

	18–29	30–39	40–49	50–59	60–69	70 +
LVEF (%)	65 (56–74)	69 (58–79)	69 (61–76)	69 (63–74)	70 (62–77)	73 (62–83)
LVEDVi (ml/m^2^)	81 (59–103)	80 (61–99)	78 (54–101)	71 (53–88)	70 (57–82)	63 (44–82)
LVESVi (ml/m^2^)	28 (17–40)	25 (16–34)	24 (13–36)	22 (14–30)	21 (14–28)	17 (7–27)
LVMi (g/m^2^)	44 (34–54)	47 (38–56)	46 (29–64)	46 (37–54)	47 (34–60)	44 (34–54)
RVEF (%)	62 (52–71)	65 (52–77)	68 (59–77)	68 (58–78)	70 (61–79)	71 (63–80)
RVEDVi (ml/m^2^)	85 (56–115)	84 (65–103)	78 (53–104)	71 (57–86)	69 (55–83)	63 (46–80)
RVESVi (ml/m^2^)	33 (16–49)	30 (19–41)	25 (11–39)	23 (14–32)	21 (12–30)	18 (12–25)
LV global longitudinal strain (%)	− 18 (− 21 to − 14)	− 18 (− 21 to − 15)	− 19 (− 23 to − 15)	− 19 (− 21 to − 16)	− 18 (− 23 to − 14)	− 19 (− 23 to − 14)
LV global radial strain (%)	30 (20–41)	32 (25–39)	35 (23–47)	33 (25–41)	33 (22–44)	35 (20–49)
LV global circumferential strain (%)	− 19 (− 24 to − 15)	− 20 (− 22 to − 19)	− 21 (− 24 to − 17)	− 21 (− 23 to − 18)	− 22 (− 26 to − 18)	− 22 (− 27 to − 17)
LAVi (ml/m^2^)*	36 (19–52)	40 (25–56)	40 (23–58)	42 (22–62)	40 (23–57)	34 (18–50)
LA strain peak (%)	20 (12.9–27.1)	21 (17.6–24.4)	22.6 (17.5–27.7)	19.7 (13.5–26)	20.2 (15.7–24.8)	18.2 (13.4–23)
LA strain conduit (%)	14.2 (7.7–20.7)	14.6 (11.7–17.4)	13.7 (10.3–17.1)	11.3 (5.9–16.8)	11 (8.4–13.6)	9.8 (6.6–13.1)
LA strain booster (%)	6.6 (2.5–10.7)	7.9 (5.1–10.6)	9.5 (6.6–12.4)	8.7 (4.8–12.5)	9.9 (6.7–13.1)	8.7 (5.4–12.1)
LV native T1 (ms)**	1215 (1194–1236)	1220 (1141–1299)	1207 (1167–1246)	1204 (1139–1269)	1204 (1150–1259)	1192 (1127–1256)
LV native T2 (ms)**	39.2 (33.9–44.5)	41.2 (37.4–45.1)	40.3 (37.5–43)	40.5 (35.1–46)	39.5 (35.6–43.5)	40.9 (37.2–44.6)
LV septal T2-star (ms)	36.7 (13.4–60)	32.1 (22.7–41.4)	32.2 (27.6–36.8)	28.3 (21–35.6)	29.6 (23–36.2)	31.6 (19–44.2)
AAo distensibility (10^−3^ mmHg^−1^)	8.9 (3.2–14.6)	7 (1.6–12.5)	4.3 (2–6.7)	3.7 (0–7.4)	2 (-0.6–4.6)	1.9 (-1.3–5)
Pulse wave velocity (m/s)	3.6 (2.3–4.8)	3.7 (2.5–5)	4.8 (2.2–7.3)	5.6 (2.7–8.4)	9.4 (1.4–17.5)	11.4 (3.5–19.2)

Mean (95% Confidence Interval)

*LA volume obtained using biplanar method

** Obtained from basal and mid slices

*AAo* Ascending Aorta, *BSA* body surface area, *g/m*^2^ grams per metre squared, *LAVi* left atrial volume index, *LVEF* left ventricular ejection fraction, *LVEDVi* left ventricular end-diastolic volume index, *LVESVi* left ventricular end-systolic volume index, *LVMi* left ventricular mass index, *ml* millilitres, *ml/m*^2^ millilitres per metre squared, *mmHg* millimetres of mercury, *m/s* metres per second, *ms* milliseconds, *RVEF* right ventricular ejection fraction, *RVEDVi* right ventricular end-diastolic volume index, *RVESVi* right ventricular end-systolic volume index

### LV strain

**Fig. 4:**
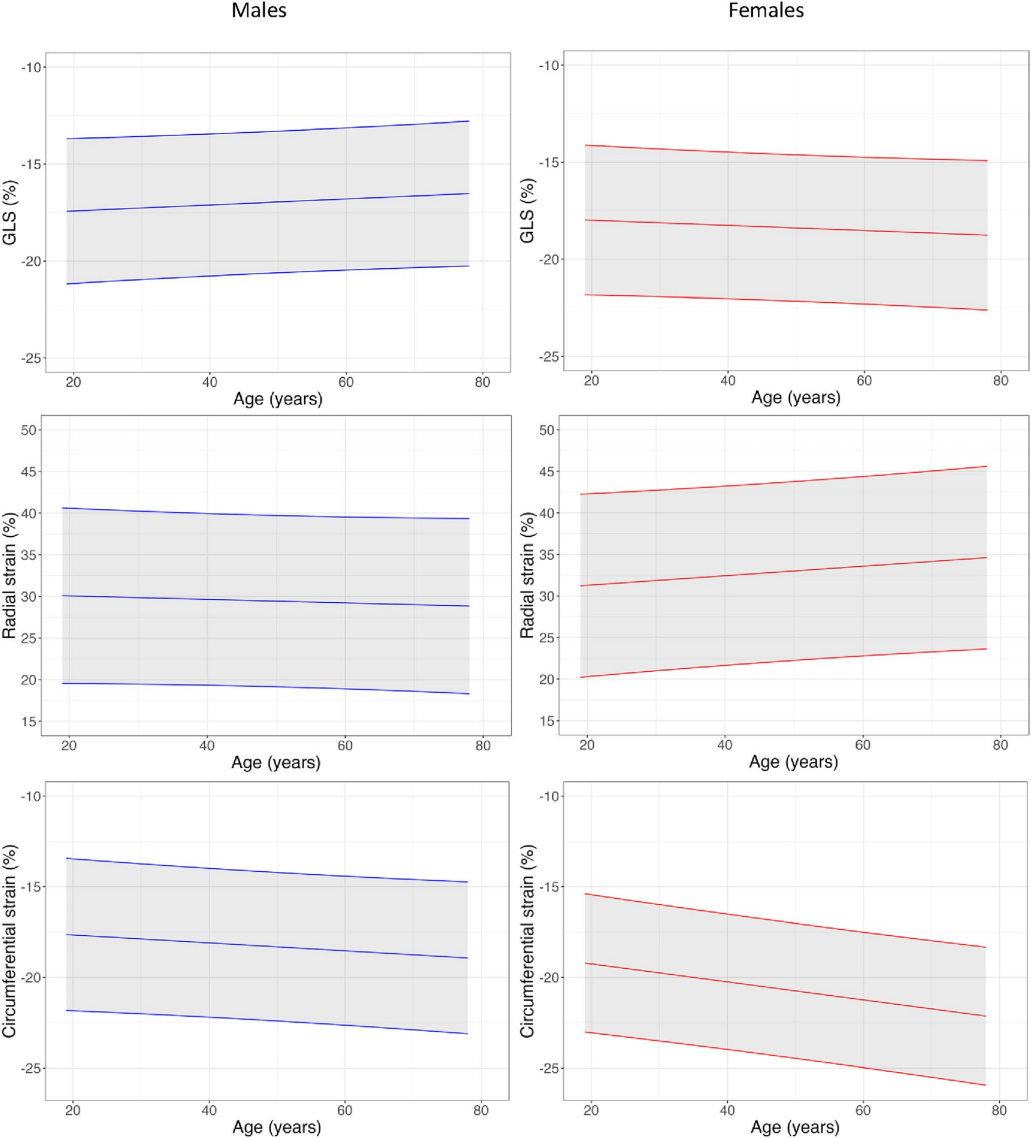
Left ventricular strain normograms

Global longitudinal, radial, and circumferential strain measurements by sex and age are presented in Tables 2, 3 and 4, Fig. 4 and Supplemental Table 4. None of the strain directions varied significantly with age for either sex except global circumferential strain, which declined with age in females. 3D strain measurements are reported in Supplemental Table 5.

**Table 4:** Reproducibility of common variations in metric assessment

	Visit 1	Visit 2	Coefficient of Variance % (95% CI)	Bland Altman Limits of Agreement
*Left Atrium*				
LA AP diameter (mm)	33 ± 5	33 ± 5	5.1 (5.0–5.3)	− 4.9, 4.7
LAAi (cm^2^/m^2^)	12 ± 3	12 ± 3	7.7 (7.5–7.9)	− 3.1, 2.6
Short axis LAVi (ml/m^2^)	46 ± 8	46 ± 7	5.6 (5.4–5.8)	− 7.3, 6.9
Biplanar LAVi (ml/m^2^)	40 ± 10	42 ± 11	12.7 (11.6–13.8)	− 17.1, 12.8
*Right Atrium*				
RAAi (cm^2^/m^2^)	12 ± 2	12 ± 2	6.4 (6.1–6.7)	− 1.9, 2.1
Short axis RAVi (ml/m^2^)	58 ± 12	58 ± 11	6.4 (6.2–6.8)	− 10.9, 10.8
*LV native T1*				
Mid slice	1186 ± 32	1187 ± 42	2.8 (2.7–2.9)	− 89.7, 88.3
Basal and mid slices	1196 ± 22	1195 ± 37	1.8 (1.76–1.83)	− 57.4, 59.2
Basal, mid and apical slices	1195 ± 25	1189 ± 44	1.9 (1.85–1.95)	− 54.2, 65.0
*LV native T2*				
Mid slice	39.5 ± 1.9	39.4 ± 2.3	3.3 (3.2–3.3)	− 3.6, 3.7
Basal and mid slices	39.0 ± 1.7	39.3 ± 2.1	2.6 (2.6–2.7)	− 3.2, 2.6
*Longitudinal strain*				
4-chamber longitudinal strain (%)	− 17.5 ± 2.0	− 17.1 ± 1.8	8.1 (7.7–8.5)	− 4.2, 3.3
Global longitudinal strain (%)	− 17.7 ± 2.1	− 17.5 ± 2.1	4.4 (4.3–4.5)	− 2.3, 1.9
3D global longitudinal strain (%)	− 11.5 ± 5.8	− 9.7 ± 9.3	36.7 (24.7–48.6)	− 11.8, 8.3

Mean ± Standard deviation

*AP* anterior posterior, *BSA* body surface area, *CI* confidence interval, *cm*^2^ centimetres squared, *cm*^2^/*m*^2^ centimetres per metre squared, *LA* left atrium, *LAAi* left atrial area index, *LAVi* left atrial volume index, *ml* millilitres, *ml/m*^2^ millilitres per metre squared, *mm* millimetres, *RAAi* right atrial area index, *RAVi* right atrial volume index, *3D* three dimensional

Longitudinal strain was similar when measured from the 4, 2 and 3 chamber cines compared to when strain was measured from the 4-chamber cine only (− 17.7 vs − 17.3%, *p* = 0.24), the former had a lower interscan reproducibility CoV (4.4 vs 8.1%) but was slower to obtain.

### Atrial size and function

**Fig. 5:**
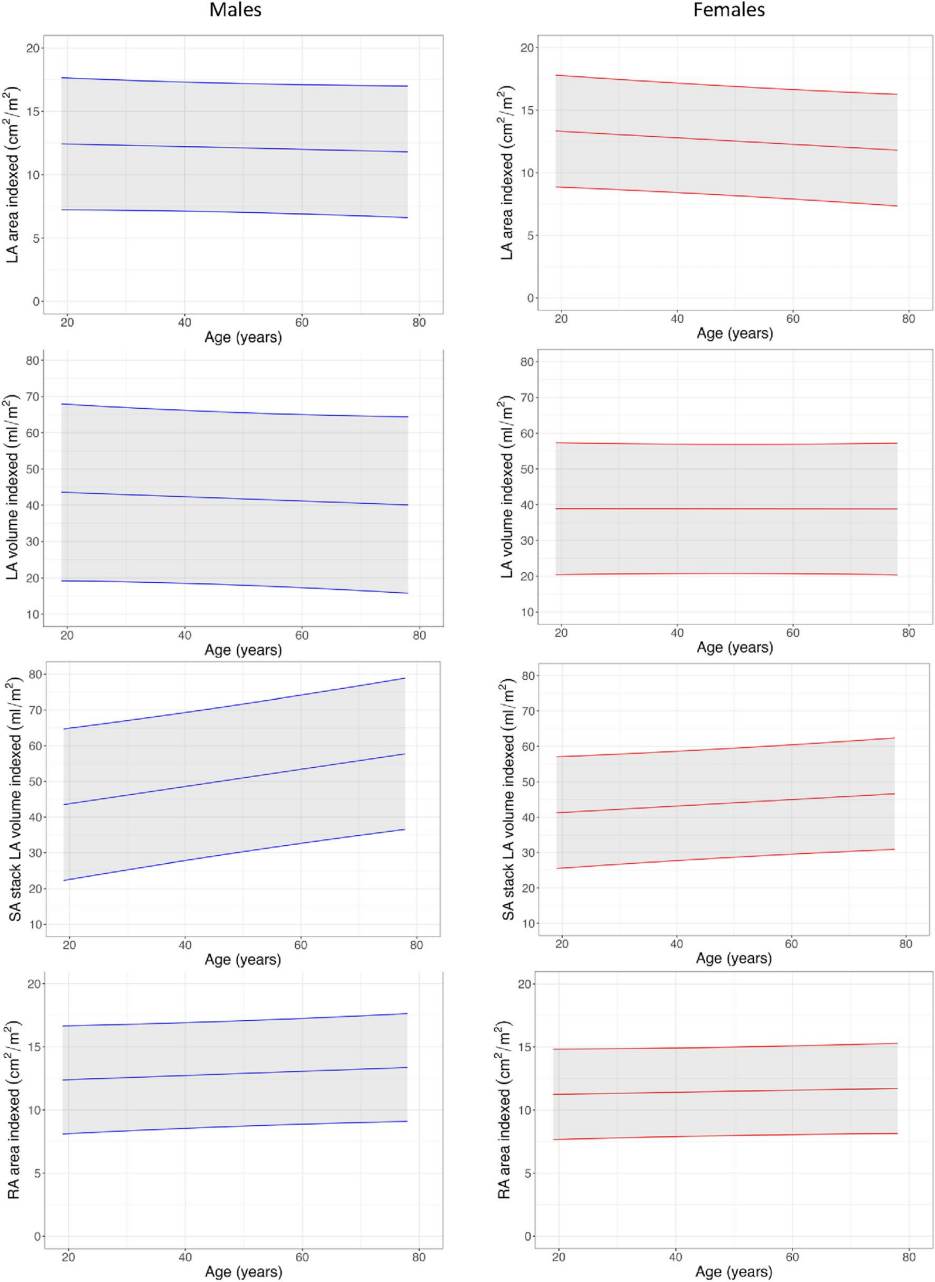
Atrial normograms

Measurements of atrial diameter, area and volumes are presented in Table 2, 3, and 4, Fig. 5 and Supplemental Table 6. Indexed atrial area and volume did not change significantly with age for either sex except for male LA volume measured using atrial short-axis images, which increased with age. LA peak and conduit strain were greater in females, but booster strain did not vary with sex. LA peak and conduit strain declined with age whereas booster strain increased.

Indexed LA volume was larger (47.3 vs 40.3 ml/m^2^, *P* < 0.0001), and its measurement more reproducible (interscan CoV = 5.6 vs 12.7%), when measured from atrial short-axis cine stacks compared with biplanar measurement from 4- and 2-chamber cines.

### Native myocardial T1, T2 and T2*

**Fig. 6:**
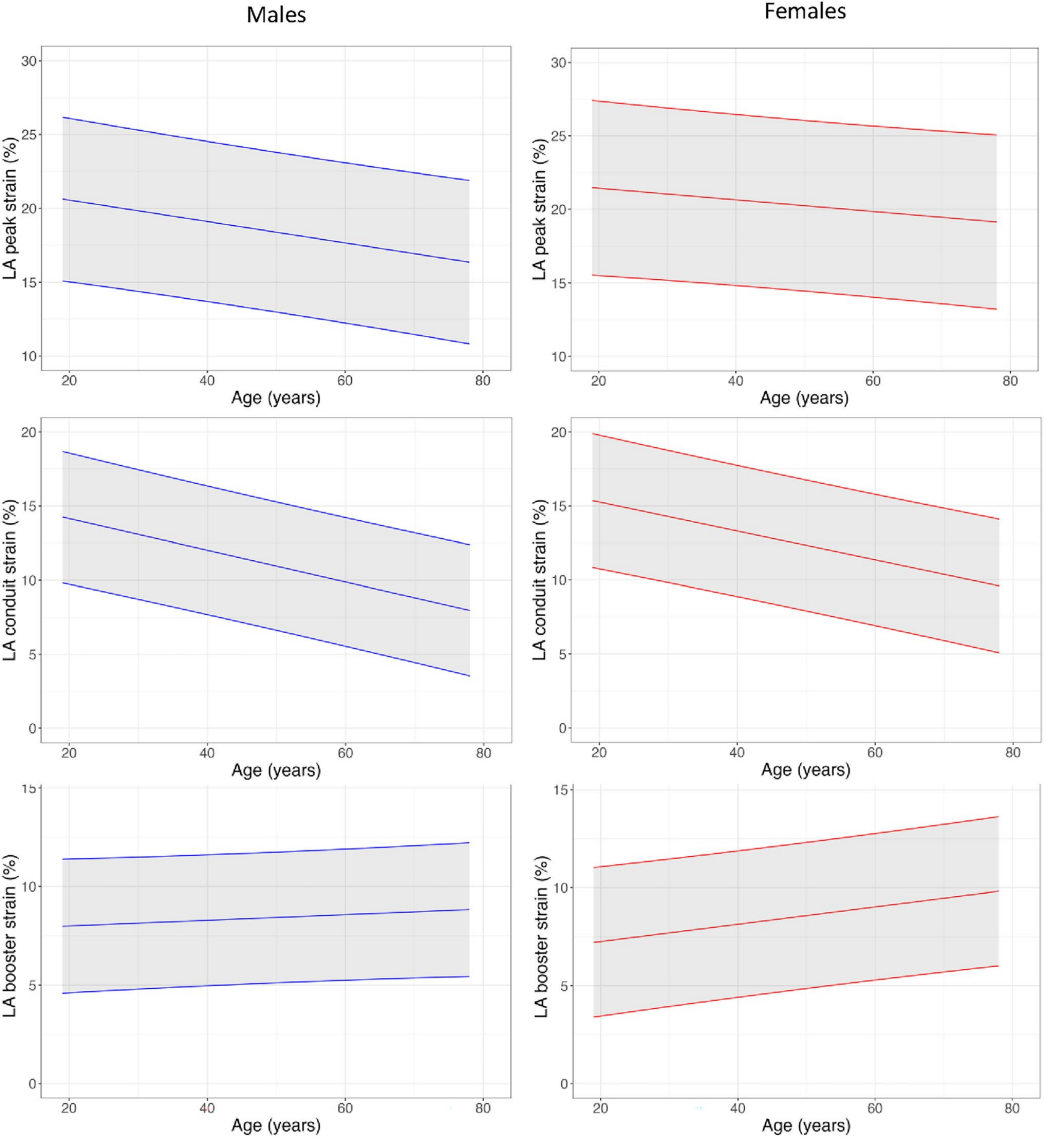
Left atrial strain normograms

Measurements of myocardial T1, T2 and T2* are presented in Tables 2, 3 and 4, Fig. 6 and Supplemental Table 7 and 8. Myocardial T1, T2 and T2* were higher in females. T1 did not change with age in males but declined significantly with age in females. T2 increased significantly with age in males but did not change with age in females. T2* decreased significantly in females with age and exhibited a trend towards reduction with age in males.

Measurements of relaxations times from basal- and mid-ventricular slices versus mid-ventricular slices only or base, mid and apical slices, and incorporating circumferential myocardium versus septum only, were associated with greater interscan reproducibility.

### Aortic distensibility and pulse wave velocity

**Fig. 7:**
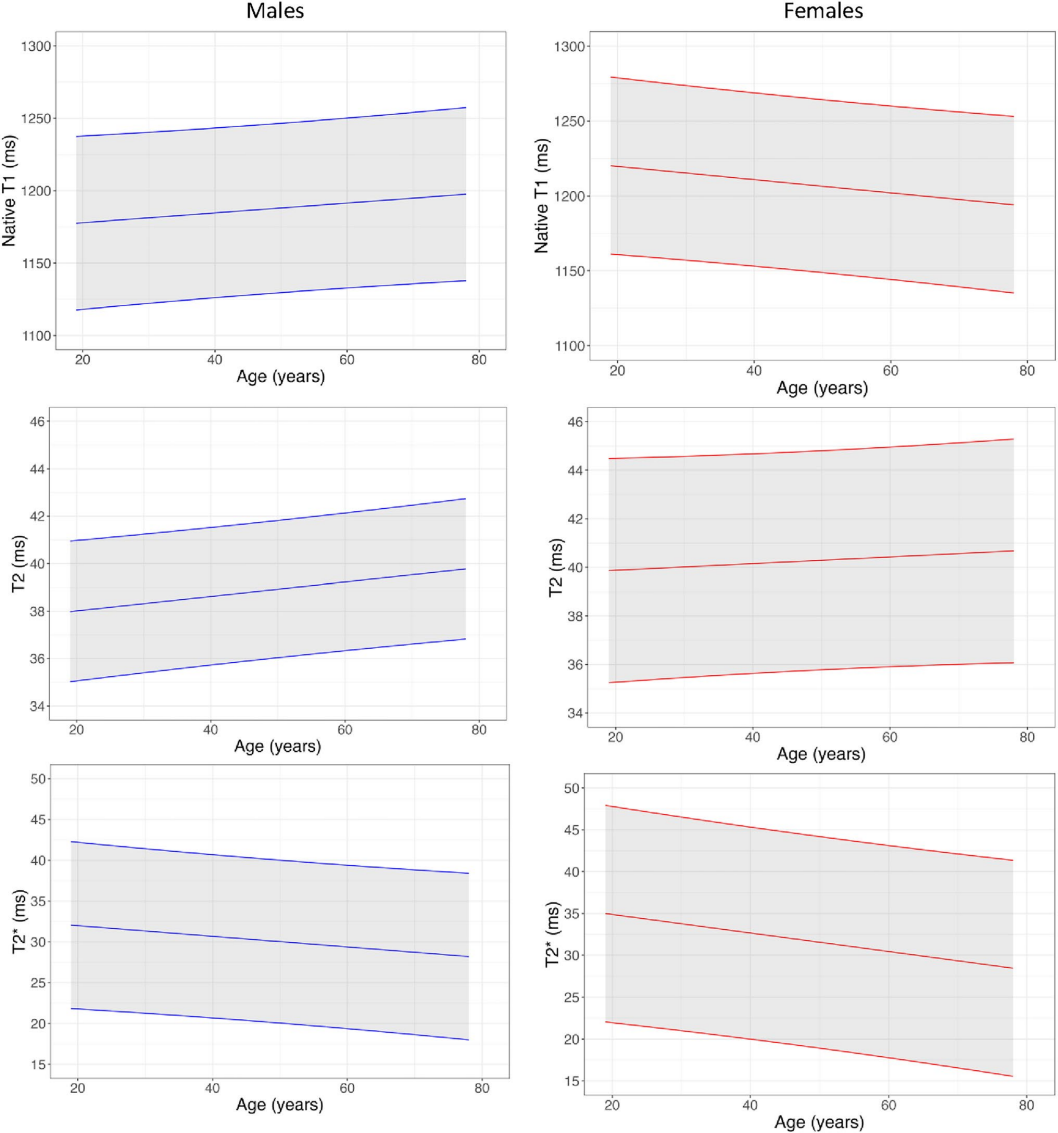
Parametric mapping normograms

**Fig. 8:**
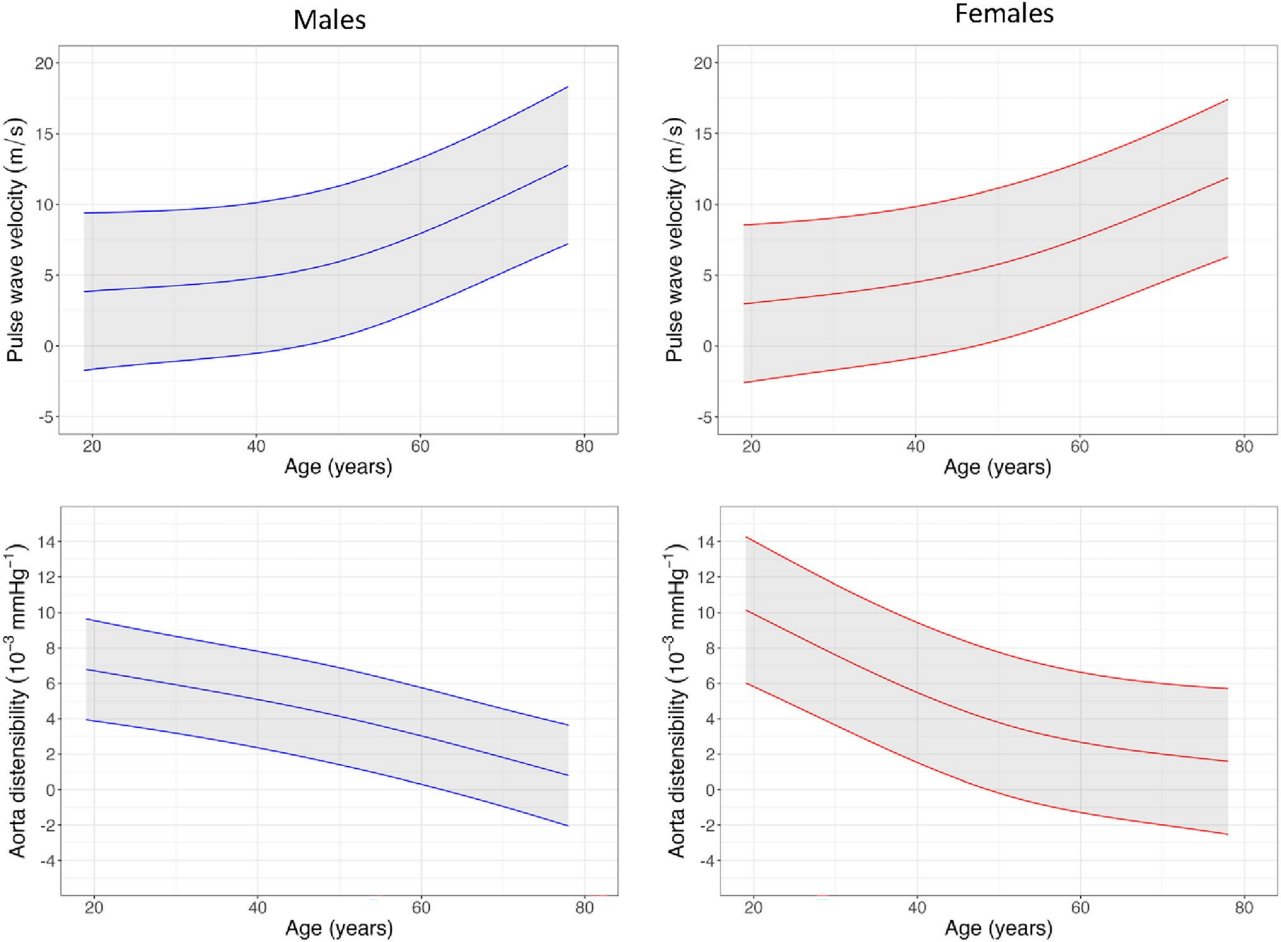
Aortic normograms

Measurements of aortic distensibility and pulse wave velocity are presented in Tables 2 and 3, Fig. 7 and Supplemental Table 9. Distensibility was higher in the ascending aorta compared to the descending aorta (4.33 vs 3.83 × 10^−3^mmhg, *P* < 0.005). Males had lower aortic distensibility than females and both sexes showed a non-linear decline with age. Similarly, males had higher pulse wave velocity and it increased with age for both sexes, with a sharp increase around the age of 50 (Fig. 8).

### Inter-scan reproducibility and inter-observer variability

Inter-scan reproducibility is presented in Table 5 and Supplemental Table 10. The reproducibility associated with common variations in how measurements are made in clinical practice are presented in Table 5. Myocardial T1 was the most reproducible of all CMR measurements, with a CoV of 1.2%. Aortic distensibility was the least reproducible measurement. The reproducibility of ventricular volumes, EF and mass was substantially higher when consistency of basal ventricular identification between baseline and follow-up scans was checked. Inter-observer variability was generally low and is presented in Table 6.

**Table 5:** Inter-scan reproducibility

	Visit 1	Visit 2	Coefficient of variance % (95% CI)	Bland Altman limits of agreement
LVEF (%)	65 ± 5	66 ± 5	4.9 (4.8–5.0)	− 10.2 to 7.7
LVEDVi (ml/m^2^)	85 ± 13	84 ± 15	4.0 (4.0–4.1)	− 7.6 to 10.5
LVESVi (ml/m^2^)	30 ± 7	29 ± 8	10.9 (10–11.7)	− 5.2 to 8.7
LVMi (g/m^2^)	55 ± 7	56 ± 9	5.1 (5.0–5.3)	− 9.2 to 6.5
RVEF (%)	61 ± 6	60 ± 5	5.2 (5.1–5.4)	− 8.1 to 9.5
RVEDVi (ml/m^2^)	92 ± 16	93 ± 18	5.4 (5.2–5.5)	− 15.6 to 13.8
RVESVi (ml/m^2^)	37 ± 10	38 ± 11	10.4 (9.8–11.1)	− 12.5 to 11.1
LV global longitudinal strain (%)	− 17.7 ± 2.1	− 17.5 ± 2.1	2.9 (2.8–2.9)	− 2.3 to 1.9
LV global radial strain (%)	30.9 ± 5.2	30.7 ± 5.3	4.1 (4.0–4.2)	− 4.9 to 5.2
LV global circumferential strain (%)	− 18.6 ± 2.4	− 19.4 ± 2.6	4.4 (4.2–4.5)	− 2.4 to 4.0
Short axis LAVi (ml/m^2^)	46 ± 8	46 ± 7	3.8 (3.7–3.9)	− 7.3 to 6.9
LA strain peak (%)	20.7 ± 5.8	20.6 ± 3.1	8.0 (7.6–8.3)	− 6.5 to 6.8
LA strain conduit (%)	13.2 ± 3.0	12.5 ± 2.6	8.6 (8.1–9.2)	− 3.3 to 4.7
LA strain booster (%)	8.6 ± 1.8	8.9 ± 1.7	10.8 (10.4–11.3)	− 4.2 to 3.5
LV native T1 (ms)	1196 ± 22	1195 ± 37	1.2 (1.2–1.2)	− 57.4 to 59.2
LV native T2 (ms)	39 ± 1.7	39.2 ± 2.1	1.8 (1.7–1.8)	− 3.2 to 2.6
LV septal T2*(ms)	31.6 ± 3.9	31.9 ± 3.7	6.8 (6.5–7.2)	− 15.6 to 18.2
AAo distensibility (10^−3^ mm Hg^−1^)	5.2 ± 2.2	5.4 ± 2.5	16.0 (14.6–17.4)	− 7.0 to 7.0
Pulse wave velocity (m/s)	5 ± 1.5	4.9 ± 1.5	7.0 (6.7–7.2)	− 1.4 to 1.4

Mean ± Standard deviation

AAo Ascending Aorta, *BSA* body surface area, *g/m*^2^ grams per metre squared, *LA* left atrium, *LAVi* left atrial volume index, *LV* left ventricle, *LVEF* left ventricular ejection fraction, *LVEDVi* left ventricular end-diastolic volume index, *LVESVi* left ventricular end-systolic volume index, *LVMi* left ventricular mass index, *ml*—millilitres, *ml/m*^2^ millilitres per metre squared, *m/s* metres per second, *ms* milliseconds, *RVEF* right ventricular ejection fraction, *RVEDVi* right ventricular end-diastolic volume index, *RVESVi* right ventricular end-systolic volume index

**Table 6:** Inter-observer variability

	Observer 1	Observer 2	Coefficient of variance % (95% CI)	Bland Altman limits of agreement
LVEF (%)	69 ± 4	67 ± 5	2.3 (2.3–2.4)	− 4.0 to 7.5
LVEDVi (ml/m^2^)	79 ± 13	81 ± 14	1.8 (1.8–1.8)	− 6.8 to 5.8,
LVESVi (ml/m^2^)	25 ± 6	27 ± 7	5.1 (5.0–5.3)	− 5.8 to 2.7
LVMi (g/m^2^)	56 ± 13	56 ± 15	5.2 (5.1–5.5)	− 11.7 to 11.8
RVEF (%)	64 ± 5	62 ± 6	3.3 (3.3–3.4)	− 6.2 to 9.8
RVEDVi (ml/m^2^)	85 ± 18	88 ± 19	2.8 (2.8–2.9)	− 11.0 to 6.4
RVESVi (ml/m^2^)	31 ± 10	34 ± 12	7.5 (7.2–7.8)	− 11.3 to 5.4
LV global longitudinal strain (%)	− 18 ± 2	− 18 ± 2	2.4 (2.4–2.5)	− 1.5 to 1.9
LV global radial strain (%)	31 ± 6	31 ± 6	3.2 (3.2–3.3)	− 4.9 to 4.0
LV global circumferential strain (%)	− 20 ± 2	− 19 ± 2	2.4 (2.4–2.5)	− 2.2 to 1.4
Short axis LAVi (ml/m^2^)	43 ± 7	45 ± 7	3.0 (3.0–3.1)	− 6.4 to 3.8
LV native T1 (ms)	1215 ± 39	1197 ± 34	0.8 (0.8–0.8)	− 2.6 to 37.4
LV native T2 (ms)	40 ± 2	40 ± 2	0.7 (0.7–0.7)	− 0.9 to 1.0

This analysis was performed independently on twenty randomly selected subjects by two observers

Mean ± Standard deviation.

*BSA* Body surface area, *g/m*^2^ grams per metre squared, *LAVi* left atrial volume index, *LV* left ventricle, *LVEF* left ventricular ejection fraction, *LVEDVi* left ventricular end-diastolic volume index, *LVESVi* left ventricular end-systolic volume index, *LVMi* left ventricular mass index, *ml* millilitres, *ml/m*^2^ millilitres per metre squared, *m/s* metres per second, *ms* milliseconds, *RVEF* right ventricular ejection fraction, *RVEDVi* right ventricular end-diastolic volume index, *RVESVi* right ventricular end-systolic volume index

## Discussion

In this study, we determine a comprehensive set of age and sex specific CMR reference ranges using contemporary acquisition protocols and analysis techniques. We show the inter-scan reproducibility of these measurements and demonstrate the impact of common variations in clinical CMR practice.

Our methodological approach followed the meticulous approach of Maceira et al. A minimum of 10 males and 10 females per age decile across a broad adult age range underwent CMR at a single centre, with careful attention paid to ensuring that patients were reliably free from known cardiovascular disease. Despite essentially identical design, the normal ranges in our study demonstrate meaningful differences compared to Maceira et al. (See Table 7 and 8). Whilst indexed normal male ranges for LV and RV ejection fraction were consistent, LV and RV volumes were higher, and LV mass was lower. Indexed female LV volumes and ejection fraction were consistent, but LV mass was substantially lower. Indexed female RV volumes were higher in the younger age groups (20–50) but lower in the older age groups; RV ejection fraction was consistent.

**Table 7:** Comparison of male normal values between studies

Measurement	Study	20–29	30–39	40–49	50–59	60–69	70 +
LVEF (%)	Farrant et al.	65 (57–72)	64 (53–76)	66 (59–73)	65 (52–77)	65 (52–78)	68 (58–78)
	Raisi-Estabragh et al. [8]	64 (51–76)	64 (52–77)	65 (53–77)	66 (53–78)	66 (54–79)	67 (55–80)
	Maceira et al. [1]	65 (57–74)	66 (57–75)	66 (58–75)	67 (58–76)	67 (58–76)	68 (59–77)
	Kawel-Boehm et al. [7]*	60 (46–74)	63 (49–77)	62 (48–76)	63 (49–78)	62 (48–76)	N/A
LVEDVi (ml/m^2^)	Farrant et al.	89 (77–101)	93 (77–108)	85 (65–104)	85 (60–111)	82 (53–111)	76 (45–106)
	Raisi-Estabragh et al. [8]	83 (58–109)	80 (55–106)	77 (51–102)	73 (48–99)	70 (45–96)	67 (41–92)
	Maceira et al. [1]	86 (68–103)	83 (66–101)	81 (64–99)	79 (62–97)	77 (60–95)	75 (58–93)
	Kawel-Boehm et al. [7]*	86 (61–112)	81 (59–103)	83 (55–110)	77 (49–105)	78 (57–99)	
LVESVi (ml/m^2^)	Farrant et al.	32 (23–41)	33 (20–46)	29 (21–36)	30 (15–46)	29 (13–46)	24 (11–38)
	Raisi-Estabragh et al. [8]	30 (17–44)	29 (15–42)	27 (13–40)	25 (12–39)	24 (10–37)	22 (9–35)
	Maceira et al. [1]	30 (19–41)	29 (18–39)	27 (17–38)	26 (15–37)	25 (14–36)	24 (13–35)
	Kawel-Boehm et al. [7]*	34 (14–53)	30 (15–46)	32 (13–50)	29 (12–45)	30 (13–46)	
LVMi (g/m^2^)	Farrant et al.	66 (44–87)	64 (50–79)	61 (41–81)	64 (49–79)	64 (43–86)	58 (43–72)
	Raisi-Estabragh et al. [8]	65 (48–81)	63 (47–80)	62 (46–79)	61 (45–78)	60 (44–77)	59 (43–76)
	Maceira et al. [1]	76 (59–93)	75 (59–92)	75 (58–91)	74 (57–91)	73 (57–90)	73 (56–89)
	Kawel-Boehm et al. [7]*	66 (44–87)	64 (41–86)	64 (43–84)	62 (42–83)	62 (38–87)	
RVEF (%)	Farrant et al.	58 (46–69)	61 (49–73)	59 (53–65)	61 (47–75)	59 (44–75)	64 (50–78)
	Raisi-Estabragh et al. [8]	56 (43–69)	57 (44–70)	58 (45–71)	58 (45–71)	59 (46–72)	59 (46–72)
	Maceira et al. [4]**	61 (48–74)	63 (50–76)	65 (52–77)	66 (53–79)	68 (55–81)	70 (57–83)
	Kawel-Boehm et al. [7]*	52 (36–69)	55 (41–68)	57 (40–73)	57 (41–74)		
RVEDVi (ml/m^2^)	Farrant et al	100 (74–125)	99 (74–124)	94 (65–123)	93 (59–127)	90 (58–122)	80 (49–111)
	Raisi-Estabragh et al. [8]	99 (70–128)	95 (66–124)	91 (62–120)	87 (58–116)	83 (54–112)	79 (50–108)
	Maceira et al. [4]**	91 (68–114)	88 (65–111)	85 (62–108)	82 (59–105)	79 (56–101)	75 (52–98)
	Kawel-Boehm et al. [7]*	94 (63–124)	83 (57–109)	81 (50–112)	80 (48–111)		
RVESVi (ml/m^2^)	Farrant et al.	43 (22–63)	40 (20–59)	39 (24–54)	38 (14–62)	37 (15–59)	29 (12–46)
	Raisi-Estabragh et al. [8]	43 (26–60)	41 (24–58)	39 (22–56)	37 (20–53)	34 (17–51)	32 (15–49)
	Maceira et al. [4]**	35 (21–50)	33 (18–47)	30 (16–45)	28 (13–42)	25 (11–40)	23 (8–37)
	Kawel-Boehm et al. [7]*	44 (23–66)	38 (22–53)	34 (18–49)	35 (16–54)		

Mean (95% Confidence Interval)

*The study by Kawel-Boehm et al. pools LV measurement data from studies by Maceira et al., Bulow et al., and Macedo et al., and pools RV measurement data from studies by Aquaro et al., Chang et al., Macedo et al.

**The study by Maceira et al. did not include papillary muscles in their right ventricular volumetric measurements

*g/m*^2^ Grams per metre squared, *LVEF* left ventricular ejection fraction, *LVEDVi* left ventricular end-diastolic volume index, *LVESVi* left ventricular end-systolic volume index, *LVMi* left ventricular mass index, *ml/m*^2^ millilitres per metre squared, *RVEF* right ventricular ejection fraction, *RVEDVi* right ventricular end-diastolic volume index, *RVESVi* right ventricular end-systolic volume index

**Table 8:** Comparison of female volumetric normative values between studies

Measurement	Study	20–29	30–39	40–49	50–59	60–69	70 +
LVEF (%)	Farrant et al	65 (56–74)	69 (58–79)	69 (61–76)	69 (63–74)	70 (62–77)	73 (62–83)
	Raisi-Estabragh et al. [8]	65 (54–76)	66 (55–77)	67 (56–78)	68 (57–79)	69 (58–80)	70 (59–81)
	Maceira et al. [1]	66 (56–75)	66 (57–75)	67 (58–76)	68 (59–77)	69 (60–78)	69 (60–78)
	Kawel-Boehm et al. [7]*	62 (50–73)	64 (52–77)	63 (50–76)	65 (52–78)	65 (53–77)	
LVEDVi (ml/m^2^)	Farrant et al	81 (59–103)	80 (61–99)	78 (54–101)	71 (53–88)	70 (57–82)	63 (44–82)
	Raisi-Estabragh et al. [8]	74 (55–94)	71 (52–91)	68 (49–88)	66 (46–85)	63 (43–82)	60 (40–79)
	Maceira et al. [1]	82 (65–99)	79 (62–96)	76 (59–93)	73 (56–90)	70 (53–87)	67 (50–84)
	Kawel-Boehm et al. [7]*	77 (54–100)	77 (52–102)	73 (50–96)	68 (48–89)	68 (51–84)	
LVESVi (ml/m^2^)	Farrant et al	28 (17–40)	25 (16–34)	24 (13–36)	22 (14–30)	21 (14–28)	17 (7–27)
	Raisi-Estabragh et al. [8]	26 (16–36)	24 (14–35)	23 (13–33)	21 (11–31)	19 (9–30)	18 (8–28)
	Maceira et al. [1]	28 (19–37)	27 (17–36)	25 (16–34)	24 (14–33)	22 (13–31)	21 (12–30)
	Kawel-Boehm et al. [7]*	29 (16–43)	29 (9–49)	27 (12–42)	24 (10–38)	25 (14–35)	
LVMi (g/m^2^)	Farrant et al	44 (34–54)	47 (38–56)	46 (29–64)	46 (37–54)	47 (34–60)	44 (34–54)
	Raisi-Estabragh et al. [8]	49 (38–61)	49 (37–61)	49 (37–60)	48 (37–60)	48 (36–60)	48 (36–59)
	Maceira et al. [1]	62 (47–77)	62 (47–77)	63 (48–77)	63 (48–78)	63 (48–78)	63 (49–78)
	Kawel-Boehm et al. [7]*	51 (29–72)	50 (32–68)	49 (32–66)	51 (31–70)	52 (31–74)	
RVEF (%)	Farrant et al	62 (52–71)	65 (52–77)	68 (59–77)	68 (58–78)	70 (61–79)	71 (63–80)
	Raisi-Estabragh et al. [8]	59 (47–71)	60 (48–72)	61 (49–73)	62 (50–74)	63 (51–75)	64 (52–76)
	Maceira et al. [4]**	61 (49–73)	63 (51–75)	65 (53–77)	67 (55–79)	69 (57–81)	71 (59–83)
	Kawel-Boehm et al. [7]*	56 (34–78)	58 (39–77)	60 (44–76)	61 (44–78)		
RVEDVi (ml/m^2^)	Farrant et al	85 (56–115)	84 (65–103)	78 (53–104)	71 (57–86)	69 (55–83)	63 (46–80)
	Raisi-Estabragh et al. [8]	82 (60–104)	79 (57–102)	76 (54–99)	74 (51–96)	71 (48–93)	68 (46–91)
	Maceira et al. [4]**	84 (65–102)	80 (61–98)	76 (57–94)	72 (53–90)	68 (49–86)	64 (45–82)
	Kawel-Boehm et al. [7]*	78 (55–101)	76 (51–100)	74 (46–102)	69 (42–95)		
RVESVi (ml/m^2^)	Farrant et al	33 (16–49)	30 (19–41)	25 (11–39)	23 (14–32)	21 (12–30)	18 (12–25)
	Raisi-Estabragh et al. [8]	33 (21–46)	32 (19–44)	30 (17–43)	28 (15–41)	26 (14–39)	25 (12–37)
	Maceira et al. [4]**	32 (20–45)	30 (17–43)	27 (14–40)	24 (11–37)	21 (8–34)	19 (6–32)
	Kawel-Boehm et al. [7]*	33 (10–56)	31 (15–48)	29 (13–45)	28 (11–44)		

Mean (95% Confidence Interval)

*The study by Kawel-Boehm et al. pools LV measurement data from studies by Maceira et al., Bulow et al., and Macedo et al., and pools RV measurement data from studies by Aquaro et al., Chang et al., Macedo et al.

**The study by Maceira et al. did not include papillary muscles in their right ventricular volumetric measurements

*g/m*^2^ grams per metre squared, *LVEF* left ventricular ejection fraction, *LVEDVi* left ventricular end-diastolic volume index, *LVESVi* left ventricular end-systolic volume index, *LVMi* left ventricular mass index, *ml/m*^2^ millilitres per metre squared, *RVEF* right ventricular ejection fraction, *RVEDVi* right ventricular end-diastolic volume index, *RVESVi* right ventricular end-systolic volume index

The reasons for these differences likely include patient population and analysis factors. The North West of England, including Greater Manchester, has the highest rates of, and worst outcomes from, cardiovascular disease in England, and a high prevalence of multiple long term conditions [11]. Life expectancy in some areas in the North West is around 20 years lower than in areas in South West England [12]. Body mass index and blood pressure in the current study were descriptively higher than that in Maceira et al. As such, while patients with known cardiovascular disease were excluded, and measurements were indexed, it is likely that ‘health’ varies according to geographical area. How healthy volunteers are recruited will also influence the population studied, and therefore potentially the measurements obtained, for example posters in a hospital versus community engagement events.

It is also likely that different CMR centres, and CMR reporters, have evolved with small differences in analysis practice. For example, how ‘hard’ to window the semi-automated thresholding tool to define the endocardium is often a personal decision based on what appears appropriate to the observer. To reduce this variability, as a centre we have adopted a method where the semi-automated thresholding is windowed to the endocardium until just before the contouring goes awry. Whilst this makes our measurements more repeatable, important for clinical trial data analysis for example, it may be a reason for the higher male ventricular volumes and lower mass observed in comparison to Maceira et al. That said, the differences in other measurements between our study and Maceira et al., including the variation between studies according to age in females, suggests population differences and potential technical differences. Such technical differences include the method of assessment for RV volumes, Maceira excluded papillary muscles which contrasts with the comparable studies in Tables 7 and 8. Maceira also used ‘CMRTools’ which is a different software package to ‘Circle CVI42’ which was used for this analysis.

The differences in normal ranges between our study and Maceira et al. are in keeping with the between study differences described by Kawel-Boehm et al. in their comprehensive review of CMR reference range data from studies published up to 2020 (Table 7 and 8). These differences raise the question of how best to define normality. On one hand, single centre data provides reference ranges specific to the population that the centre serves, which as highlighted earlier, may be important. On the other hand, different centres having different reference ranges impacts data transferability (would a normal range for LVEDVi of up to 111 ml/m^2^ for men aged 50–70 as defined in our centre be accepted in other centres?), what is the impact of patients moving geographical location, and saliently, few centres have the required scanning time, and person time (e.g., for research ethics applications etc.), to define their centre’s normal ranges through a study such as this.

The recent study by Raisi-Estabragh et al. (Healthy Hearts Consortium) provides a different approach. Raisi-Estabragh et al. defined normal ranges for common CMR variables using data from more than 9000 CMR scans from six studies in Europe and Asia (85% of scans were from UK Biobank). This approach overcomes many of the disadvantages of single centre data, and has resulted in very robust normal ranges. It has also enabled normal ranges to be defined for a range of ethnicities, which is critically important, and which is difficult for any single centre to perform. They also highlight the subtle variations which can arise from magnet field strength and vendor which cannot be accounted for in single centre studies.

In addition to the volumetric data, this study provides a comprehensive set of normal ranges for other parameters such LV strain, atrial volumes and strain, native T1, T2, T2*, aortic distensibility and pulse wave velocity. There was no change in indexed left atrial volume with healthy ageing however there was a decrease in atrial conduit strain and an increase in atrial booster strain suggesting an increase in atrial stiffness with advancing age. In this population there was a sex influence on values of T1 and T2 with both being higher in females and there were sex specific changes in myocardial T1 and T2 with healthy aging. Measures of vascular aging such as proximal aortic distensibility are independent predictors of mortality and cardiovascular events [13]. In this study distensibility decreased and pulse wave velocity increased with advancing age which is in keeping with other studies [14].

Our study also serves to demonstrate the impact of common variations in clinical CMR practice, highlighting the need for corresponding reference ranges. For example, LA size is a strong prognostic indicator, is usually included in clinical reports and is increasingly used as a clinical trial endpoint. In the current study, indexed LA volume measured significantly larger (7 ml/m^2^) from short axis atrial stack images that from biplanar measurement from 4- and 2-chamber cines. Measuring myocardial T1 from a mid-ventricular slice compared to basal and mid-ventricular slices resulted in reduced interscan reproducibility, suggesting that a basal and mid slice may be required where appropriate.

Interscan reproducibility for common CMR measurements was in keeping with previous published data [15, 16]. Whilst not a new observation, it is worth noting for clinical practice that, for example, the Bland Altman 95% limits of agreement show that measurement of LVEF can be expected to vary by up to around 18% (approximately ± 9%) between CMR scans. When assessing for incremental change it is good practice to look back on previous scan contours to ensure consistency, our data shows the impact this has on reproducibility, and the value in doing this. We demonstrate that when follow-up scans are analysed by the same observer after checking that basal ventricular identification is consistent with the baseline scan, measurement reproducibility is much higher, e.g., approximately ± 4.5%. Automated artificial intelligence analysis is undoubtedly the way forward, removing interobserver variability [15]. Nevertheless, when interval scans are analysed by a single experienced observer, with between-scan reference to ensure consistency of basal ventricular identification, our data show that measurement reproducibility is similar to that of current automated techniques [15].

## Limitations

Normal MCMR is limited as a single centre study. Despite recruiting a patient population free from known cardiovascular disease, we are unable to account for regional variations in population anthropomorphic, clinical, and ethnic characteristics. Such variation is reflected in the higher body mass index and blood pressure when compared to other studies. The single centre nature of the study also precluded assessment using anything other than a Siemens 3 T scanner. Secondly, whilst we report the association of variables with factors such as age, the study was not powered for this purpose and all such analyse should be considered exploratory. Finally, despite the inter-observer variability being in keeping with other published studies, the normal reference ranges are established from analyses performed by a single observer which is a limitation.

## Conclusion

This study provides a comprehensive set of age and sex specific CMR reference ranges, along with inter-scan reproducibility, and describes the impact of common variations in practice. Single centre studies such as this, whilst meticulous in design and delivery, result in clinically-relevant differences in normal ranges. We advocate that larger cohorts, including diverse ethnicities, such as the Healthy Hearts Consortium, may represent a better approach to defining normal ranges for common CMR measurements.

## Supplementary Information

Below is the link to the electronic supplementary material.

Supplementary file1 (DOCX 84 KB)

## Data Availability

All data used to support the results and conclusion of this study is included within the manuscript and supplemental appendix. Requests for additional data should be submitted to the corresponding author.

## Abbreviations

2D: Two-dimensional
3D: Three-dimensional
AAo: Ascending Aorta
AP: Anterior–posterior
BMI: Body mass index
BP: Blood pressure
BSA: Body surface area
CoV: Coefficient of variation
CMR: Cardiovascular magnetic resonance
EF: Ejection fraction
LA: Left atrial
LAVi: Left atrial volume index
LV: Left ventricular
LVEDVi: Left ventricular end-diastolic volume index
LVESVi: Left ventricular end-systolic volume index
LVEF: Left ventricular ejection fraction
LVMi: Left ventricular mass index
RAVi: Right atrial volume index
RV: Right ventricular
RVEDVi: Right ventricular end-diastolic volume index
RVESVi: Right ventricular end-systolic volume index
RVEF: Right ventricular ejection fraction
TAPSE: Tricuspid annular plane systolic excursion
