# Supplementary material for: Comprehensive reference ranges for cardiovascular magnetic resonance: time to move on from single centre data?
Source: Int J Cardiovasc Imaging. 2025 Mar 27;41(5):913–32. doi: 10.1007/s10554-025-03370-5 (PMC12075261; doi:10.1007/s10554-025-03370-5)
Supplement: Supplementary file 1 — Supplementary file1 (DOCX 84 KB) [file 10554_2025_3370_MOESM1_ESM.docx]

**Comprehensive reference ranges for cardiovascular magnetic resonance: time to move on from single centre data?**

**Supplemental Appendix**

Table of Contents

CMR acquisition protocol 3

CMR image analysis 5

Ventricular mass, volumes and ejection fraction 5

Left ventricular strain 5

Atrial volumes and strain 6

Parametric mapping 6

Aortic distensibility and pulse wave velocity 7

Supplemental Table 1. Baseline characteristics 8

Supplemental Table 2. Left ventricular volumetrics and ejection fraction 9

Supplemental Table 3. Right ventricular volumetrics and ejection fraction 10

Supplemental Table 4. Left ventricular 2D strain 11

Supplemental Table 5. Left ventricular 3D strain 12

Supplemental Table 6. Atrial volumes and strain 13

Supplemental Table 7. Parametric Imaging 15

Supplemental Table 8. Condensed normal ranges for parametric mapping. 16

Supplemental Table 9. Great vessels 17

Supplemental Table 10 Ventricular Volumetric Reproducibility with basal slice checking. 18

Supplemental table 11a Linear associations of key male metrics with age 19

Supplemental table 11b Linear associations of key female metrics with age 20

Supplemental References 21

# CMR acquisition protocol

1. Localisers to derive the cardiac planes as per local procedure e.g., 2-chamber localiser, 4-chamber localiser, short-axis localiser.
2. 4-chamber balanced steady state free-precession (bSSFP) breath-hold cine.
3. 2-chamber bSSFP breath-hold cine.
4. 3-chamber bSSFP breath-hold cine.
5. LVOT long-axis (i.e. perpendicular to the 3-chamber cine) bSSFP breath-hold cine.
6. RVOT bSSFP breath-hold cine.
7. RV in and out bSSFP breath-hold cine.
8. Cine perpendicular to the ascending and descending aorta at pulmonary bifurcation level (40 phases). Measure BP during acquisition.
9. Phase encoded velocity mapping of the aorta perpendicular to the ascending and descending aorta at pulmonary bifurcation level i.e. copy image position from the step above. VENC 1.5 m/s. (40 phases).
10. Ventricular short-axis bSSFP breath-hold cine stack.

8mm slices. 2mm inter-slice gap.

Check each slice for artefact and repeat as necessary.

Check to ensure the base and apex of the ventricles are fully covered.

1. Magnetisation transfer weighted bSSFP cine with differing flip angles and RF pulse durations.
2. Basal, mid and apical LV short-axis T1 mapping. Modified Look-Locker Inversion Recovery (MOLLI).

- Basal slice position: Copy to the most basal short-axis cine slice that does not contain any LVOT at end-diastole i.e. there should be a ‘complete ring’ of myocardium.
- Mid slice position: Copy to the short-axis cine slice that is two slices more apical than the slice chosen as the basal slice.
- Apical slice position: Copy to the short-axis cine slice that is two slices more apical than the slice chosen as the mid slice.

1. Basal and mid LV short-axis T2 mapping.

- Basal slice position: Copy to the most basal short-axis cine slice that does not contain any LVOT at end-diastole i.e. there should be a ‘complete ring’ of myocardium.
- Mid slice position: Copy to the short-axis cine slice that is two slices more apical than the slice chosen as the basal slice.

1. Mid LV short-axis T2* mapping.

- Mid slice position: Copy the mid slice position used for the T2 map.

1. Atrial short-axis bSSFP breath-hold cine stack.

6mm slices. No inter-slice gap.

Check each slice for artefact and repeat as necessary.

# CMR image analysis

## Ventricular mass, volumes and ejection fraction

All volumetric, mass, functional and wall thickness data were measured from bSSFP breath-hold cine images in accordance with recommendations from Society for Cardiovascular Magnetic Resonance (SCMR).^1^ End-diastole and end-systole were identified for each slice of the bSSFP short-axis ventricular stack and LV endocardial and epicardial contours along with the anterior and inferior right ventricular insertion points were defined using a semi-automated thresholding tool to define detailed contours, post-hoc freehand correction was performed as needed. Papillary muscles and trabecular tissue were included in measurement of LV mass (See Figure 1).

## Left ventricular strain

LV strain values were measured using semi-automated feature tracking analysis. The 2D and 3D algorithms have been described previously.^2,3^ Briefly, the 2D feature tracking algorithm determines myocardial deformation using reference points in the mid myocardial wall, which are tracked in all phases. These points are derived from the diastolic LV endo- and epi-cardial contours. Throughout the cardiac cycle positions of these reference points move and can be tracked by surrounding features in two directions, thus yielding independent 2D motion fields for short- and long- axis data. 3D feature tracking relies on a 3D deformation model generated by combining the 2D image information into a single 3D field, motions within the field are used to quantify strain either globally or segmentally.

In this study, the SSFP short axis ventricular stack and 4-,2- and 3-chamber long axis bSSFP cines each including 25 phases were analysed. End-diastole was identified and LV endocardial and epicardial contours along with the anterior and inferior right ventricular insertion points were defined in all slices. Strain analyses were automatically performed by feature tracking software in all slices containing both endocardial and epicardial contours. In cases of inaccurate tracking, LV contours were manually adjusted post hoc, and the algorithm reapplied. This generated 2D/3D strain curves and a 16-segment polar map.  To determine the optimal method of assessment 2D global longitudinal strain was calculated using two methods. Firstly, by contouring the 4-chamber SSFP breath hold cine image only and secondly using the 4,2 and 3-chamber SSFP breath hold cine images.

## Atrial volumes and strain

LA diameters were measured antero-posteriorly from the 3 chamber bSSFP cine in atrial diastole. Atrial areas were measured in atrial diastole using the 4 chamber SSFP cine. LA volumes were measured using two methods: 1. LA length, mitral annular diameter and endocardial borders were contoured from the 4- and 2-chamber bSSFP cine images and volume was calculated using the Simpson’s biplane method; 2. Endocardial borders were contoured using LA short-axis bSSFP cine images and volume was then calculated using the Simpson’s summation of discs method.

LA strain values were measured using feature tracking as described previously.^4^ Atrial diastole was identified using 4-,2- and 3-chamber long axis SSFP cines and LA endocardial and epicardial contours were defined. Strain curves were generated using feature tracking software. Contours were manually adjusted, and the algorithm reapplied in cases of inaccurate tracking.

## Parametric mapping

T1 and T2 relaxation time was assessed using a variety of different methods designed to represent the variations seen in clinical practice. T1 maps were analysed in the septum and circumferentially using a single mid ventricular slice, basal and mid ventricular slice and a basal, mid and apical slice. T2 maps were analysed in the septum and circumferentially using a single mid ventricular slice and a basal and mid ventricular slice.

T1 and T2 maps were autogenerated by the Siemens 3T Vida scanner using a vendor supplied MOLLi and T2-prep bSSFP, respectively. LV endocardial and epicardial contours along with the anterior and inferior right ventricular insertion points were defined in all slices. A 30% endocardial and epicardial offset was then applied to ensure that only the middle 40% of myocardium on the T1 and T2 maps were included in the measurement of native myocardial T1 and T2 relaxation times. The included myocardium was segmented into thirds automatically by the software which allowed assessment of septal T1 and T2 values. To obtain T2* a region of interest was drawn involving the middle third the LV septum on raw T2* images to obtain the T2* time.

## Aortic distensibility and pulse wave velocity

Aortic distensibility was obtained by measuring the maximal and minimal aortic area (Amax and Amin) from a single slice bSSFP cine at the level of the pulmonary artery bifurcation. The pulse pressure (PP) calculated as the difference between systolic and diastolic pressure was obtained during the scan at the time of acquisition. Distensibility was then calculated using the following formula: (Amax-Amin)/(Amin×PP) in mm Hg^−1^. To calculate the pulse wave velocity (PWv), through-plane flow measurements at the level of the pulmonary trunk cutting across both the ascending and the proximal descending aorta were obtained. The ascending and descending aorta were contoured to produce corresponding flow maps. PWv was subsequently calculated using a dedicated calculation tool which uses the distance along the aortic centerline between measurement locations (obtained from bSSFP cine imaging) and the foot-to-foot transit time from the resulting velocity waveforms.

# Supplemental Table 1. Baseline characteristics

Mean ± standard deviation

| **Males** | **18-29** | **30-39** | **40-49** | **50-59** | **60-69** | **70+** |
| --- | --- | --- | --- | --- | --- | --- |
| Height (cm) | 179±7 | 180±5 | 174±8 | 179±8 | 175±8 | 174±8 |
| Weight (Kg) | 80±16 | 81±12 | 77±14 | 88±21 | 83±9 | 83±16 |
| BSA | 1.99±0.23 | 2.01±0.16 | 1.93±0.2 | 2.08±0.26 | 2±0.15 | 2±0.21 |
| BMI | 24.9±3.6 | 25±3.3 | 25.5±3.5 | 27.5±5 | 27.1±2.1 | 27.3±4.2 |
| Heart rate | 71±12 | 61±8 | 63±6 | 63±14 | 68±14 | 70±13 |
| Systolic BP | 121±19 | 117±7 | 112±8 | 120±10 | 139±17 | 140±22 |
| Diastolic BP | 65±5 | 66±8 | 68±5 | 73±11 | 81±12 | 72±9 |
| PR Interval | 153±23 | 160±15 | 165±15 | 174±23 | 173±21 | 168±30 |
| QRS Duration | 101±8 | 94±6 | 96±13 | 101±7 | 94±11 | 101±10 |
| QTc Interval | 416±18 | 398±19 | 407±21 | 411±30 | 420±16 | 420±21 |
| Axis | 38±31 | 49±40 | 23±36 | 26±30 | -7±38 | 4±23 |
| **Females** | **18-29** | **30-39** | **40-49** | **50-59** | **60-69** | **70+** |
| Height (cm) | 165±8 | 165±8 | 165±6 | 162±4 | 161±7 | 157±7 |
| Weight (Kg) | 61±12 | 72±19 | 69±14 | 64±9 | 60±9 | 68±11 |
| BSA | 1.67±0.2 | 1.8±0.27 | 1.78±0.18 | 1.69±0.12 | 1.64±0.16 | 1.72±0.17 |
| BMI | 22.1±3.4 | 26±5.8 | 25.4±5.3 | 24.6±3.4 | 23.1±2.6 | 27.9±4 |
| Heart rate | 80±20 | 69±10 | 65±7 | 69.7±8.1 | 73±8 | 68±10 |
| Systolic BP | 104±7 | 104±7 | 117±11 | 117±17 | 128±19 | 127±16 |
| Diastolic BP | 60±7 | 62±5 | 69±8 | 68±11 | 66±10 | 64±10 |
| PR Interval | 148±31 | 140±25 | 150±13 | 151±13 | 159±21 | 173±41 |
| QRS Duration | 89±5 | 91±8 | 91±9 | 97±11 | 92±7 | 89±5 |
| QTc Interval | 417±21 | 419±25 | 422±19 | 422±13 | 419±21 | 427±16 |
| Axis | 72±20 | 47±39 | 44±27 | 37±38 | 35±18 | 11±30 |

Abbreviations: BMI – body mass index, BP – blood pressure, BSA – body surface area, cm – centimetres, Kg – kilograms.

# Supplemental Table 2. Left ventricular volumetrics and ejection fraction

Mean, 95% Confidence Interval

| **Males** | **18-29** | **30-39** | **40-49** | **50-59** | **60-69** | **70+** |
| --- | --- | --- | --- | --- | --- | --- |
| LVEDV (ml) | 182(117-246) | 192(137-246) | 168(107-229) | 186(125-246) | 169(99-239) | 153(79-227) |
| LVESV (ml) | 64(34-95) | 66(39-92) | 57(38-77) | 63(29-97) | 59(26-93) | 49(19-79) |
| LVEF (%) | 65(56-74) | 66(54-77) | 66(59-73) | 67(55-78) | 65(54-76) | 68(60-77) |
| LVSV (ml) | 118(72-163) | 126(83-170) | 111(65-156) | 123(83-163) | 110(67-152) | 104(57-151) |
| LVM (g) | 132(68-196) | 130(86-174) | 118(65-170) | 133(101-165) | 128(81-176) | 116(73-158) |
| LV papillary mass (g)* | 3.8(1-7) | 4.4(1-8) | 3.1(1-5) | 3.5(2-5) | 2.9(1-5) | 2.5(1-4) |
| LVEDVi (ml/m^2^) | 91(77-105) | 95(78-113) | 87(64-110) | 90(61-119) | 84(53-116) | 76(46-106) |
| LVESVi (ml/m^2^) | 32(22-42) | 33(19-46) | 30(21-38) | 30(16-44) | 30(12-47) | 24(11-38) |
| LVSVi (ml/m^2^) | 59(46-72) | 62(49-76) | 57(40-74) | 60(37-82) | 55(38-71) | 52(33-70) |
| LVMi (g/m^2^) | 66(45-87) | 64(50-79) | 61(41-81) | 64(50-79) | 64(42-86) | 58(43-72) |
| LV maximal wall thickness (mm) | 11(7-14) | 11(8-13) | 11(9-13) | 12(10-13) | 12(10-14) | 12(11-13) |
| **Females** | **18-29** | **30-39** | **40-49** | **50-59** | **60-69** | **70+** |
| LVEDV (ml) | 137(80-193) | 144(103-184) | 140(97-183) | 123(87-159) | 115(84-145) | 109(76-142) |
| LVESV (ml) | 48(22-74) | 45(23-68) | 42(23-61) | 39(24-53) | 35(23-46) | 30(12-48) |
| LVEF (%) | 65(56-74) | 69(59-79) | 70(63-77) | 69(63-74) | 70(62-77) | 73(62-85) |
| LVSV (ml) | 89(53-124) | 98(73-123) | 98(69-126) | 84(60-109) | 80(56-105) | 79(57-102) |
| LVM (g) | 73(48-99) | 85(53-117) | 83(46-120) | 77(59-95) | 78(47-108) | 75(57-94) |
| LV papillary mass (g)* | 1.8(1-3) | 2.2(0-4) | 1.8(1-3) | 1.7(1-3) | 1.6(0-3) | 1.5(0-3) |
| LVEDVi (ml/m^2^) | 81(59-103) | 80(60-101) | 79(53-105) | 72(56-88) | 70(56-84) | 64(46-81) |
| LVESVi (ml/m^2^) | 28(17-40) | 25(16-34) | 24(14-34) | 23(15-30) | 21(14-28) | 17(7-27) |
| LVSVi (ml/m^2^) | 53(38-68) | 55(38-73) | 55(37-74) | 50(39-60) | 49(39-58) | 46(34-59) |
| LVMi (g/m^2^) | 44(34-54) | 47(38-56) | 47(29-64) | 45(36-55) | 47(34-60) | 44(34-54) |
| LV maximal wall thickness (mm) | 8(7-10) | 9(6-11) | 9(6-12) | 10(7-12) | 10(8-12) | 11(8-13) |

*****Papillary mass is included in the calculation of overall LV mass.

Abbreviations: BSA – body surface area, g – grams, g/m2 – grams per metre squared, LV – left ventricle, LVEF - left ventricular ejection fraction, LVEDV - left ventricular end-diastolic volume, LVEDVi - left ventricular end-diastolic volume index, LVESV – left ventricular end-systolic volume, LVESVi - left ventricular end-systolic volume index, LVM - left ventricular mass, LVMi - left ventricular mass index, LVSV - left ventricular stroke volume, LVSVi - left ventricular stroke volume index, ml - millilitres, ml/m2 - millilitres per metre squared, mm – millimetres.

# Supplemental Table 3. Right ventricular volumetrics and ejection fraction

Mean, 95% Confidence Interval

| **Males** | **18-29** | **30-39** | **40-49** | **50-59** | **60-69** | **70+** |
| --- | --- | --- | --- | --- | --- | --- |
| RVEDV (ml) | 203(104-302) | 205(137-273) | 182(109-256) | 198(128-269) | 184(108-260) | 162(86-237) |
| RVESV (ml) | 86(28-144) | 79(38-120) | 73(38-108) | 75(33-118) | 74(29-120) | 58(20-96) |
| RVSV (ml) | 118(71-164) | 126(82-170) | 109(64-155) | 123(84-162) | 110(68-152) | 104(55-153) |
| RVEF (%) | 59(48-70) | 62(50-74) | 60(52-68) | 63(51-74) | 60(46-74) | 64(50-78) |
| RVEDVi (ml/m^2^) | 101(72-130) | 102(78-125) | 94(66-123) | 97(55-138) | 92(59-124) | 81(51-110) |
| RVESVi (ml/m^2^) | 42(22-63) | 39(20-58) | 38(22-53) | 37(13-60) | 37(15-59) | 29(12-45) |
| RVSVi (ml/m^2^) | 59(45-72) | 62(49-76) | 56(39-74) | 60(37-82) | 55(38-71) | 52(33-71) |
| TAPSE (mm) | 22.9(12.7-33.1) | 21(16-26) | 21(11-31) | 19(12-26) | 20(15-25) | 18(12-25) |
| **Females** | **18-29** | **30-39** | **40-49** | **50-59** | **60-69** | **70+** |
| RVEDV (ml) | 144(78-209) | 151(108-194) | 141(93-189) | 122(87-157) | 114(80-147) | 110(79-140) |
| RVESV (ml) | 55(23-88) | 54(25-83) | 44(18-70) | 38(22-55) | 34(18-50) | 31(18-43) |
| RVSV (ml) | 89(53-124) | 97(71-124) | 97(69-125) | 84(58-110) | 80(55-104) | 79(56-102) |
| RVEF (%) | 62(53-72) | 65(52-78) | 69(60-79) | 69(59-79) | 70(61-79) | 72(63-81) |
| RVEDVi (ml/m^2^) | 86(56-115) | 85(65-104) | 80(51-109) | 72(57-86) | 69(55-83) | 64(46-82) |
| RVESVi (ml/m^2^) | 33(16-49) | 30(19-41) | 25(11-39) | 22(14-31) | 21(12-30) | 18(10-26) |
| RVSVi (ml/m^2^) | 53(38-68) | 55(36-74) | 55(37-74) | 49(38-60) | 48(39-57) | 46(33-59) |
| TAPSE (mm) | 22.1(16.3-27.9) | 23.4(14.6-32.2) | 20.9(14.5-27.3) | 20.5(16.9-24.1) | 20.6(14.7-26.5) | 18.3(10.3-26.3) |

Abbreviations: BSA – body surface area, g – grams, g/m2 – grams per metre squared, ml - millilitres, ml/m2 - millilitres per metre squared, mm – millimetres, RVEDV - right ventricular end-diastolic volume, RVEDVi - right ventricular end-diastolic volume index, RVEF - right ventricular ejection fraction, RVESV - right ventricular end-systolic volume, RVESVi - right ventricular end-systolic volume index, TAPSE – tricuspid annular plane systolic excursion.

# Supplemental Table 4. Left ventricular 2D strain

Mean, 95% Confidence Interval

| **Males** | **18-29** | **30-39** | **40-49** | **50-59** | **60-69** | **70+** |
| --- | --- | --- | --- | --- | --- | --- |
| Global longitudinal strain (%) | -17(-22--13) | -17(-20--15) | -17(-19--15) | -17(-22--12) | -16(-20--13) | -17(-21--13) |
| Global radial strain (%) | 31(19-42) | 30(23-37) | 29(24-33) | 29(16-43) | 28(19-37) | 30(18-42) |
| Global circumferential strain (%) | -18(-22--14) | -18(-22--15) | -18(-20--15) | -18(-23--12) | -18(-20--15) | -20(-24--17) |
| 4-chamber longitudinal strain (%) | -17(-22--11) | -16(-19--13) | -17(-21--12) | -17(-21--13) | -17(-21--13) | -18(-25--11) |
| **Females** | **18-29** | **30-39** | **40-49** | **50-59** | **60-69** | **70+** |
| Global longitudinal strain (%) | -18(-21--14) | -18(-21--15) | -19(-23--15) | -19(-21--16) | -18(-23--14) | -19(-23--14) |
| Global radial strain (%) | 30(20-41) | 32(25-39) | 35(23-47) | 33(25-41) | 33(22-44) | 35(20-49) |
| Global circumferential strain (%) | -19(-24--15) | -20(-22--19) | -21(-24--17) | -21(-23--18) | -22(-26--18) | -22(-27--17) |
| 4-chamber longitudinal strain (%) | -18(-20--15) | -17(-21--14) | -19(-24--14) | -18(-21--15) | -18(-24--11) | -18(-23--12) |

Abbreviations: 2D – two dimensional.

# Supplemental Table 5. Left ventricular 3D strain

| **Males** | **18-29** | **30-39** | **40-49** | **50-59** | **60-69** | **70+** |
| --- | --- | --- | --- | --- | --- | --- |
| 3D global longitudinal strain (%) | -11(-18--3) | -12(-17--8) | -11(-15--7) | -10(-20-0) | -6(-19-6) | -10(-17--4) |
| 3D basal longitudinal strain (%) | 2(-28-32) | -6(-17-4) | -4(-15-6) | 1(-18-21) | 10(-17-36) | 2(-17-22) |
| 3D mid longitudinal strain (%) | -10(-17--3) | -11(-16--6) | -10(-15--5) | -9(-22-3) | -3(-30-23) | -8(-23-7) |
| 3D apical longitudinal strain (%) | -17(-22--12) | -17(-20--14) | -17(-20--13) | -17(-21--12) | -16(-19--12) | -17(-21--13) |
| 3D global radial strain (%) | 34(19-49) | 33(13-52) | 32(15-49) | 33(15-51) | 36(21-50) | 41(8-73) |
| 3D basal radial strain (%) | 55(0-110) | 45(20-70) | 45(24-67) | 48(9-87) | 51(28-74) | 59(16-103) |
| 3D mid radial strain (%) | 31(0-63) | 34(22-47) | 31(20-43) | 34(16-51) | 42(-3-87) | 42(-19-103) |
| 3D apical radial strain (%) | 27(-6-60) | 27(7-48) | 28(-2-59) | 27(14-40) | 30(21-38) | 35(13-56) |
| 3D global circumferential strain (%) | -18(-27--10) | -19(-23--15) | -19(-23--15) | -19(-24--13) | -18(-22--15) | -20(-23--16) |
| 3D basal circumferential strain (%) | -11(-35-12) | -15(-19--12) | -15(-20--11) | -15(-23--7) | -15(-20--9) | -15(-33-3) |
| 3D mid circumferential strain (%) | -19(-25--13) | -19(-23--15) | -18(-23--14) | -18(-24--13) | -18(-21--15) | -19(-23--15) |
| 3D apical circumferential strain (%) | -23(-30--15) | -23(-28--18) | -23(-28--17) | -23(-28--18) | -22(-27--17) | -23(-28--19) |
| **Females** | **18-29** | **30-39** | **40-49** | **50-59** | **60-69** | **70+** |
| 3D global longitudinal strain (%) | -13(-17--8) | -13(-17--9) | -12(-27-4) | -14(-18--10) | -15(-19--10) | -14(-20--9) |
| 3D basal longitudinal strain (%) | 2(-45-48) | -7(-17-3) | -4(-28-20) | -8(-16-0) | -7(-24-9) | -3(-27-21) |
| 3D mid longitudinal strain (%) | -12(-18--5) | -12(-17--6) | -9(-35-16) | -14(-19--9) | -14(-21--8) | -14(-19--10) |
| 3D apical longitudinal strain (%) | -17(-21--14) | -18(-20--16) | -19(-24--13) | -18(-21--16) | -19(-23--15) | -19(-23--16) |
| 3D global radial strain (%) | 43(26-60) | 40(34-46) | 44(19-70) | 41(31-52) | 44(31-58) | 47(25-70) |
| 3D basal radial strain (%) | 58(33-83) | 60(42-77) | 57(23-91) | 61(30-93) | 57(33-81) | 61(7-114) |
| 3D mid radial strain (%) | 40(26-53) | 39(29-49) | 47(-9-104) | 38(29-47) | 40(31-50) | 46(13-78) |
| 3D apical radial strain (%) | 38(13-63) | 30(19-41) | 38(8-68) | 35(20-49) | 42(14-70) | 58(-21-137) |
| 3D global circumferential strain (%) | -20(-25--16) | -21(-24--19) | -20(-39-0) | -22(-24--20) | -23(-26--19) | -22(-29--15) |
| 3D basal circumferential strain (%) | -9(-45-27) | -18(-20--15) | -14(-40-11) | -18(-22--14) | -19(-23--15) | -16(-34-2) |
| 3D mid circumferential strain (%) | -20(-25--16) | -21(-24--18) | -18(-44-7) | -22(-24--20) | -22(-26--18) | -22(-28--17) |
| 3D apical circumferential strain (%) | -25(-31--19) | -25(-30--21) | -26(-32--20) | -26(-29--24) | -26(-30--23) | -26(-34--18) |

Abbreviations: 3D – three dimensional**.**

# Supplemental Table 6. Atrial volumes and strain

Mean, 95% Confidence Interval

| **Males** | **18-29** | **30-39** | **40-49** | **50-59** | **60-69** | **70+** |
| --- | --- | --- | --- | --- | --- | --- |
| **Left atrium** |  |  |  |  |  |  |
| LA AP diameter (mm) | 32(23-40) | 35(27-42) | 35(27-42) | 39(27-51) | 39(23-56) | 37(25-49) |
| LAA (cm^2^) | 24.2(14.8-33.6) | 26(16.1-35.9) | 24.2(15.5-33) | 23(13.7-32.4) | 23.5(9.4-37.5) | 24(12.9-35) |
| LAAi (cm^2^/m^2^) | 12.2(7.8-16.6) | 12.9(9.5-16.2) | 12.6(8.9-16.2) | 11.3(5.5-17.1) | 11.7(5.3-18.1) | 12.1(6.3-17.8) |
| Short axis LAV (ml) | 88(44-132) | 98(58-139) | 95(59-131) | 112(76-148) | 108(48-168) | 109(51-166) |
| Short axis LAVi (ml/m^2^) | 44(28-59) | 49(35-62) | 49(35-63) | 55(31-79) | 54(27-80) | 54(28-81) |
| Biplanar LAV (ml) | 83(27-138) | 95(47-142) | 84(52-117) | 83(55-111) | 74(7-141) | 83(25-140) |
| Biplanar LAVi (ml/m^2^) | 41(18-64) | 47(28-65) | 44(30-57) | 41(22-59) | 37(5-68) | 42(13-71) |
| LA longitudinal strain peak (%) | 19.3(14.3-24.2) | 20.5(15.3-25.6) | 19.7(15.2-24.2) | 17.2(11.9-22.5) | 18.3(14.2-22.5) | 15.7(9.8-21.6) |
| LA longitudinal strain conduit (%) | 13.5(8.4-18.7) | 12.6(9.8-15.3) | 12.2(8-16.3) | 10.3(5.2-15.4) | 9.4(5.9-12.9) | 8.3(3.6-13) |
| LA longitudinal strain booster (%) | 7.3(4.9-9.7) | 9(5.4-12.7) | 8.7(4.2-13.1) | 8(5.5-10.6) | 9.3(7.1-11.5) | 8.2(5.6-10.7) |
| **Right atrium** |  |  |  |  |  |  |
| RAA (cm^2^) | 23.9(12.1-35.6) | 27.3(16.8-37.9) | 23.8(16.7-30.9) | 28.5(19.1-37.8) | 25.5(17-34) | 25.7(19.6-31.9) |
| RAAi (cm^2^/m^2^) | 11.9(7.7-16) | 13.5(10-17.1) | 12.4(9.3-15.4) | 13.9(8-19.7) | 12.7(9.4-16) | 12.9(9.4-16.5) |
| RAV (ml) | 133(48-218) | 135(61-209) | 124(70-178) | 134(61-207) | 118(57-179) | 115(50-180) |
| RAVi (ml/m^2^) | 66(35-97) | 66(40-93) | 64(42-86) | 66(23-109) | 58(33-84) | 58(27-89) |
| **Females** | **18-29** | **30-39** | **40-49** | **50-59** | **60-69** | **70+** |
| **Left atrium** |  |  |  |  |  |  |
| LA AP diameter (mm) | 29(18-40) | 32(25-40) | 34(24-43) | 32(23-40) | 32(22-43) | 35(25-46) |
| LAA (cm^2^) | 21.4(14.3-28.5) | 21.9(18.6-25.3) | 23(15.8-30.3) | 22.6(16.6-28.6) | 20.5(12.4-28.6) | 18.8(9.9-27.7) |
| LAAi (cm^2^/m^2^) | 12.9(8.9-16.9) | 12.4(8.8-15.9) | 13.1(8.5-17.7) | 13.4(9.2-17.5) | 12.5(8.2-16.8) | 10.9(6.6-15.2) |
| Short axis LAV (ml) | 67(32-101) | 78(57-100) | 80(47-112) | 78(50-107) | 73(53-93) | 76(41-112) |
| Short axis LAVi (ml/m^2^) | 40(23-56) | 44(31-57) | 45(28-62) | 46(32-61) | 45(35-54) | 44(25-63) |
| Biplanar LAV (ml) | 60(27-93) | 71(51-92) | 71(40-103) | 71(35-107) | 66(31-101) | 59(27-91) |
| Biplanar LAVi (ml/m^2^) | 36(19-52) | 40(25-56) | 40(23-58) | 42(22-62) | 40(23-57) | 34(18-50) |
| LA longitudinal strain peak (%) | 20(12.9-27.1) | 21(17.6-24.4) | 22.6(17.5-27.7) | 19.7(13.5-26) | 20.2(15.7-24.8) | 18.2(13.4-23) |
| LA longitudinal strain conduit (%) | 14.2(7.7-20.7) | 14.6(11.7-17.4) | 13.7(10.3-17.1) | 11.3(5.9-16.8) | 11(8.4-13.6) | 9.8(6.6-13.1) |
| LA longitudinal strain booster (%) | 6.6(2.5-10.7) | 7.9(5.1-10.6) | 9.5(6.6-12.4) | 8.7(4.8-12.5) | 9.9(6.7-13.1) | 8.7(5.4-12.1) |
| **Right atrium** |  |  |  |  |  |  |
| RAA (cm^2^) | 19.6(11.4-27.8) | 19.6(11.2-28) | 19.6(11.2-28) | 19.6(11.2-28) | 19.6(11.2-28) | 19.6(11.2-28) |
| RAAi (cm^2^/m^2^) | 11.7(8.2-15.2) | 11.9(9.5-14.2) | 10.4(6.6-14.1) | 11.2(8.7-13.6) | 11.9(9.5-14.4) | 11.9(7-16.9) |
| RAV (ml) | 89(39-138) | 102(64-140) | 88(53-123) | 89(62-116) | 89(64-113) | 79(51-107) |
| RAVi (ml/m^2^) | 53(28-77) | 57(33-81) | 50(30-69) | 52(38-67) | 54(40-68) | 46(32-60) |

AP – anterior posterior, BSA – body surface area, cm^2^ – centimetres squared, cm^2^/m^2^ – centimetres per metre squared, LA – left atrium, LAA - left atrial area, LAAi - left atrial area index, LAV - left atrial volume, LAVi - left atrial volume index, ml - millilitres, ml/m2 - millilitres per metre squared, mm – millimetres, RAA - right atrial area, RAAi – right atrial area index, RAV - right atrial volume, RAVi –right atrial volume index.

# Supplemental Table 7. Parametric Imaging

Mean, 95% Confidence Interval

| **Males** | | **20-29** | **30-39** | **40-49** | **50-59** | **60-69** | **70+** |
| --- | --- | --- | --- | --- | --- | --- | --- |
| LV native T1 | Mid slice | 1174(1133-1215) | 1184(1153-1215) | 1170(1128-1212) | 1182(1127-1237) | 1168(1075-1261) | 1185(1093-1277) |
|  | Basal and mid slices | 1177(1138-1217) | 1190(1168-1213) | 1177(1135-1220) | 1194(1144-1243) | 1190(1119-1261) | 1197(1103-1291) |
|  | Basal, mid and apical slices | 1170(1136-1205) | 1186(1162-1209) | 1170(1136-1205) | 1187(1133-1241) | 1179(1112-1246) | 1190(1085-1295) |
| LV native septal T1 | Mid slice | 1205(1160-1250) | 1214(1169-1259) | 1197(1147-1248) | 1208(1161-1256) | 1189(1078-1299) | 1207(1091-1324) |
|  | Basal and mid slices | 1205(1163-1246) | 1220(1195-1244) | 1203(1163-1243) | 1218(1173-1262) | 1214(1126-1303) | 1219(1108-1330) |
|  | Basal, mid and apical slices | 1196(1158-1233) | 1210(1188-1231) | 1194(1151-1236) | 1208(1161-1256) | 1198(1112-1283) | 1207(1072-1343) |
| LV native T2 | Mid slice | 38.8(35.3-42.4) | 38.4(34.8-42.1) | 39.4(37.2-41.5) | 39.7(36.5-42.8) | 39.1(3.05-43.2) | 40.1(37.7-42.5) |
|  | Basal and mid slices | 38.4(35.3-41.4) | 38.2(35.2-41.1) | 38.8(36.9-40.7) | 39.4(36.8-42.1) | 38.9(35.4-42.3) | 39.8(36.8-42.8) |
| LV native septal T2 | Mid slice | 40.3(35.7-44.9) | 39.1(34.9-43.2) | 39.6(37.6-41.6) | 40.1(36.7-43.6) | 39.6(35.2-44.0) | 40.8(38.5-43.1) |
|  | Basal and mid slices | 39.1(35.7-42.6) | 38.7(35.4-41.9) | 39.2(37.9-40.6) | 39.7(37.0-42.4) | 39.5(35.6-43.4) | 40.3(37.9-42.7) |
| LV septal T2-star | Mid slice | 31.0(24.0-38.0) | 31.6(24.3-38.9) | 30.2(23.7-36.7) | 31.6(16.8-46.5) | 28.0(20.5-35.5) | 28.2(15.6-40.8) |
| **Females** | | **20-29** | **30-39** | **40-49** | **50-59** | **60-69** | **70+** |
| LV native T1 | Mid slice | 1209(1160-1258) | 1217(1128-1305) | 1205(1166-1245) | 1198(1125-1270) | 1196(1135-1257) | 1179(1102-1257) |
|  | Basal and mid slices | 1215(1194-1236) | 1220(1141-1299) | 1207(1167-1246) | 1204(1139-1269) | 1204(1150-1259) | 1192(1127-1256) |
|  | Basal, mid and apical slices | 1226(1202-1251) | 1226(1162-1290) | 1215(1169-1261) | 1218(1108-1329) | 1210(1135-1285) | 1195(1116-1274) |
| LV native septal T1 | Mid slice | 1232(1197-1267) | 1247(1162-1332) | 1227(1176-1279) | 1231(1161-1302) | 1215(1146-1283) | 1216(1140-1291) |
|  | Basal and mid slices | 1240(1217-1264) | 1250(1176-1324) | 1228(1191-1265) | 1234(1161-1308) | 1222(1159-1285) | 1216(1151-1281) |
|  | Basal, mid and apical slices | 1246(1220-1272) | 1247(1169-1325) | 1231(1183-1278) | 1241(1125-1356) | 1228(1142-1313) | 1217(1149-1285) |
| LV native T2 | Mid slice | 38.9(33.3-44.5) | 41.6(37.5-45.7) | 40.9(37.3-44.4) | 40.8(33.7-48.0) | 39.7(35.5-43.9) | 40.9(37.7-44.1) |
|  | Basal and mid slices | 39.2(33.9-44.5) | 41.2(37.4-45.1) | 40.3(37.5-43.0) | 40.5(35.1-46.0) | 39.5(35.6-43.5) | 40.9(37.2-44.6) |
| LV native septal T2 | Mid slice | 39.8(33.4-46.2) | 42.4(37.3-47.6) | 40.9(37.4-44.3) | 40.9(35.2-46.5) | 40.0(35.1-44.8) | 40.8(37.9-43.7) |
|  | Basal and mid slices | 40.2(36.1-44.3) | 41.9(36.8-47.1) | 40.4(37.8-43.0) | 40.8(35.8-45.9) | 39.8(35.8-43.8) | 40.7(37.5-44.0) |
| LV septal T2-star | Mid slice | 36.7(13.4-60.0) | 32.1(22.7-41.4) | 32.2(27.6-36.8) | 28.3(21.0-35.6) | 29.6(23.0-36.2) | 31.6(19.0-44.2) |

Abbreviations: LV – left ventricle.

# Supplemental Table 8. Condensed normal ranges for parametric mapping.

Mean (95% Confidence Interval).

|  | **Male** | **Female** | **Total** |
| --- | --- | --- | --- |
| LV native T1 (ms)* | 1188(1130-1245) | 1207(1149-1265) | 1197(1137-1258) |
| LV native T2 (ms)* | 38.9(35.9-41.9) | 40.3(35.9-44.7) | 39.6(35.6-43.6) |
| LV septal T2*(ms) | 30.1(20.2-40) | 31.6(18.9-44.4) | 30.9(19.4-42.4) |

* Obtained from basal and mid slices.

Abbreviations: LV – left ventricle, ms - milliseconds.

# Supplemental Table 9. Great vessels

Mean, 95% Confidence Interval

| **Males** | **18-29** | **30-39** | **40-49** | **50-59** | **60-69** | **70+** |
| --- | --- | --- | --- | --- | --- | --- |
| Pulmonary artery diameter (mm) | 22(17-27) | 23(18-28) | 24(18-29) | 23(18-29) | 24(21-28) | 23(19-27) |
| Ascending aorta distensibility (10^−3^ mm Hg^−1^) | 6.3(3.6-9.1) | 5.6(2.5-8.7) | 4.4(1.2-7.5) | 4.5(1.4-7.7) | 1.7(0.6-2.9) | 1.6(0-3.3) |
| Descending aorta distensibility (10^−3^ mm Hg^−1^) | 4.8(1.1-8.4) | 4.7(2.3-7.1) | 4.2(0.1-8.4) | 3.9(1.5-6.3) | 2.2(-0.1-4.6) | 1.8(-0.4-3.9) |
| Pulse wave velocity (m/s) | 4.1(2.6-5.7) | 4.4(3-5.8) | 5.2(2.7-7.6) | 6.3(0.8-11.8) | 9.6(1.8-17.4) | 11.3(2.9-19.6) |
| Time to 50% peak ascending aortic flow (ms) | 57.4(23.7-91) | 56.7(31.6-81.9) | 65.2(48.5-82) | 69.6(40.6-98.5) | 67.9(36.1-99.8) | 48(13-83.1) |
| Time to 50% peak descending aortic flow (ms) | 81(50.7-111.3) | 81.3(61.5-101) | 87.2(68-106.4) | 91(57.2-124.8) | 83.9(51.2-116.5) | 62.8(30-95.6) |
| **Females** | **18-29** | **30-39** | **40-49** | **50-59** | **60-69** | **70+** |
| Pulmonary artery diameter (mm) | 21(16-25) | 22(18-25) | 22(17-27) | 21(15-27) | 22(17-27) | 21(16-26) |
| Ascending aorta distensibility (10^−3^ mm Hg^−1^) | 8.9(3.2-14.6) | 7(1.6-12.5) | 4.3(2-6.7) | 3.7(0-7.4) | 2(-0.6-4.6) | 1.9(-1.3-5) |
| Descending aorta distensibility (10^−3^ mm Hg^−1^) | 6.2(2.7-9.6) | 5.8(1-10.7) | 4.2(0.1-8.4) | 3.5(1.6-5.3) | 2.4(0.3-4.4) | 2.3(-0.2-4.9) |
| Pulse wave velocity (m/s) | 3.6(2.3-4.8) | 3.7(2.5-5.0) | 4.8(2.2-7.3) | 5.6(2.7-8.4) | 9.4(1.4-17.5) | 11.4(3.5-19.2) |
| Time to 50% peak ascending aortic flow (ms) | 56.3(28.3-84.3) | 60.4(46.4-74.5) | 63.1(31.4-94.7) | 63.5(40.1-86.8) | 56.9(28-85.9) | 58.5(37.8-79.2) |
| Time to 50% peak descending aortic flow (ms) | 82.4(51.1-113.6) | 87.1(71.6-102.6) | 84.8(59.3-110.2) | 83.9(54.4-113.5) | 71(44.3-97.7) | 71.2(46.9-95.4) |

Abbreviations: mm – millimetres, mmHg – millimetres of mercury, m/s – metres per second, ms – milliseconds.

# Supplemental Table 10 Ventricular Volumetric Reproducibility with basal slice checking.

|  |  | **Visit 1** | **Visit 2** | **Coefficient of Variance % (95% CI)** | **Bland Altman Limits of Agreement** |
| --- | --- | --- | --- | --- | --- |
| LVEF (%) | | 65±5 | 67±5 | 1.7 (1.7-1.8) | -5.3, 3.9 |
| LVEDVi (ml/m^2^) | | 85±13 | 86±15 | 2.5 (2.5-2.6) | -10.1, 8.8 |
| LVESVi (ml/m^2^) | | 30±7 | 29±8 | 4.5 (4.4-4.6) | -5.2, 5.9 |
| LVMi (g/m^2^) | | 55±7 | 55±9 | 2.9 (2.9-3.0) | -6.8, 7.2 |
| RVEF (%) | | 61±6 | 63±6 | 3.0 (2.9-3.0) | -8.3, 7.0 |
| RVEDVi (ml/m^2^) | | 92±16 | 91±17 | 2.7 (2.6-2.7) | -10.5, 11.3 |
| RVESVi (ml/m^2^) | | 37±10 | 35±10 | 6.3 (6.1-6.6) | -8.1, 10.5 |

Mean ± Standard deviation

Abbreviations: BSA - body surface area, CI - confidence interval, g/m2 - grams per metre squared, LVEF - left ventricular ejection fraction, LVEDVi - left ventricular end-diastolic volume index, LVESVi - left ventricular end-systolic volume index, LVMi - left ventricular mass index, ml/m2 - millilitres per metre squared, RVEF - right ventricular ejection fraction, RVEDVi - right ventricular end-diastolic volume index, RVESVi - right ventricular end-systolic volume index.

# Supplemental table 11a Linear associations of key male metrics with age

|  | **Linear association with age** | |
| --- | --- | --- |
|  | **β (95% CI)** | **p** |
| LVEF (%) | 0.05(-0.03 to 0.12) | 0.19 |
| LVEDVi (ml/m^2^) | -0.27(-0.07 to -0.46) | 0.008 |
| LVESVi (ml/m^2^) | -0.13(-0.22 to -0.03) | 0.01 |
| LVMi (g/m^2^) | -0.07(-0.21 to 0.06) | 0.28 |
| RVEF (%) | 0.06(-0.03 to 0.15) | 0.19 |
| RVEDVi (ml/m^2^) | -0.33(-0.57 to -0.09) | 0.008 |
| RVESVi (ml/m^2^) | -0.19(-0.04 to -0.34) | 0.01 |
| LV GLS (%) | 0.02(-0.01 to 0.04) | 0.26 |
| LV GRS (%) | -0.02(-0.1 to 0.06) | 0.58 |
| LV GCS (%) | -0.02(-0.05 to 0.01) | 0.16 |
| LAVi (ml/m^2^)* | -0.05(-0.23 to 0.11) | 0.5 |
| LA strain peak (%) | -0.07(-0.11 to -0.03) | <0.005 |
| LA strain conduit (%) | -0.11(-0.13 to -0.07) | <0.005 |
| LA strain booster (%) | 0.01 (-0.01 to 0.04) | 0.25 |
| LV native T1 (ms)** | 0.4 (-0.1-0.8) | 0.12 |
| LV native T2 (ms)** | 0.03 (0.01-0.05) | 0.006 |
| LV septal T2-star (ms) | -0.1 (-0.1-0.0) | 0.08 |
| AA distensibility (10^−3^ mmHg^−1^) | -0.26(-0.34 to -0.17) | <0.005 |
| Pulse wave velocity (m/s) | 0.15 (0.11 to 0.19) | <0.005 |

*LA volume obtained using biplanar method.

** Obtained from basal and mid slices.

Abbreviations – AA – Ascending Aorta, BSA - body surface area, CI - confidence interval, g/m2 - grams per metre squared, LV GCS – left ventricular global circumferential strain, LV GLS - Left ventricular global longitudinal strain, LV GRS - Left ventricular global radial strain, LAVi - BSA indexed left atrial volume, LVEF - left ventricular ejection fraction, LVEDVi - BSA indexed LV end-diastolic volume, LVESVi - BSA indexed LV end-systolic volume, LVMi - BSA indexed LV mass, ml - millilitres, ml/m2 - millilitres per metre squared, mmHg - millimetres of mercury, m/s - metres per second, ms - milliseconds, RVEF - right ventricular ejection fraction, RVEDVi - BSA indexed right ventricular end-diastolic volume, RVESVi - BSA indexed right ventricular end-systolic volume.

# Supplemental table 11b Linear associations of key female metrics with age

|  | **Linear association with age** | |
| --- | --- | --- |
|  | **β (95% CI)** | **p** |
| LVEF (%) | 0.12(0.05 to 0.18) | <0.005 |
| LVEDVi (ml/m^2^) | -0.32(-0.47 to -0.17) | <0.005 |
| LVESVi (ml/m^2^) | -0.18(-0.25 to -0.1) | <0.005 |
| LVMi (g/m^2^) | 0.01(-0.08 to 0.09) | 0.84 |
| RVEF (%) | 0.17(0.09 to 0.24) | <0.005 |
| RVEDVi (ml/m^2^) | -0.41(-0.57 to -0.25) | <0.005 |
| RVESVi (ml/m^2^) | -0.26(-0.35 to -0.17) | <0.005 |
| LV GLS (%) | -0.01(-0.04 to 0.01) | 0.33 |
| LV GRS (%) | 0.06(-0.02 to 0.14) | 0.15 |
| LV GCS (%) | -0.05(-0.08 to -0.02) | <0.005 |
| LAVi (ml/m^2^)* | 0.0 (-0.13 to 0.13) | 0.98 |
| LA strain peak (%) | -0.04(-0.08 to 0.0) | 0.06 |
| LA strain conduit (%) | -0.09(-0.12 to -0.07) | <0.005 |
| LA strain booster (%) | 0.04(0.02 to 0.07) | <0.005 |
| LV native T1 (ms)** | -0.44(-0.86 to -0.02) | 0.03 |
| LV native T2 (ms)** | 0.01(-0.02 to 0.05) | 0.4 |
| LV septal T2-star (ms) | -0.11(-0.2 to -0.02) | 0.02 |
| AA distensibility (10^−3^ mmHg^−1^) | -0.21(-0.27 to -0.15) | <0.005 |
| Pulse wave velocity (m/s) | 0.15(0.11 to 0.19) | <0.005 |

*LA volume obtained using biplanar method.

** Obtained from basal and mid slices.

Abbreviations – AA – Ascending Aorta, BSA - body surface area, g/m2 - grams per metre squared, LV GCS – left ventricular global circumferential strain, LV GLS - Left ventricular global longitudinal strain, LV GRS - Left ventricular global radial strain, LAVi - BSA indexed left atrial volume, LVEF - left ventricular ejection fraction, LVEDVi - BSA indexed LV end-diastolic volume, LVESVi - BSA indexed LV end-systolic volume, LVMi - BSA indexed LV mass, ml - millilitres, ml/m2 - millilitres per metre squared, mmHg - millimetres of mercury, m/s - metres per second, ms - milliseconds, RVEF - right ventricular ejection fraction, RVEDVi - BSA indexed right ventricular end-diastolic volume, RVESVi - BSA indexed right ventricular end-systolic volume.

# Supplemental References

1. Schulz-Menger J, Bluemke DA, Bremerich J, et al. Standardized image interpretation and post-processing in cardiovascular magnetic resonance - 2020 update : Society for Cardiovascular Magnetic Resonance (SCMR): Board of Trustees Task Force on Standardized Post-Processing. J Cardiovasc Magn Reson 2020;22(1):19. DOI: 10.1186/s12968-020-00610-6.

2. Fröjdh F, Fridman Y, Bering P, et al. Extracellular Volume and Global Longitudinal Strain Both Associate With Outcomes But Correlate Minimally. JACC Cardiovasc Imaging 2020;13(11):2343-2354. (In eng). DOI: 10.1016/j.jcmg.2020.04.026.

3. Liu B, Dardeer AM, Moody WE, et al. Reference ranges for three-dimensional feature tracking cardiac magnetic resonance: comparison with two-dimensional methodology and relevance of age and gender. Int J Cardiovasc Imaging 2018;34(5):761-775. (In eng). DOI: 10.1007/s10554-017-1277-x.

4. Nayyar D, Nguyen T, Pathan F, et al. Cardiac magnetic resonance derived left atrial strain after ST-elevation myocardial infarction: an independent prognostic indicator. Cardiovasc Diagn Ther 2021;11(2):383-393. DOI: 10.21037/cdt-20-879.
